# Supplementary material for: Arabidopsis thaliana and Pseudomonas Pathogens Exhibit Stable Associations over Evolutionary Timescales
Source: Cell Host Microbe. 2018 Jul 11;24(1):168–179.e4. doi: 10.1016/j.chom.2018.06.011 (PMC6054916; doi:10.1016/j.chom.2018.06.011)
Supplement: Document S1. Figures S1–S6 and Tables S1 and S2 [file mmc1.pdf]

Cell Host & Microbe, Volume *24*

Supplemental Information

Arabidopsis thaliana and *Pseudomonas* Pathogens Exhibit Stable Associations over Evolutionary Timescales

Talia L. Karasov, Juliana Almario, Claudia Friedemann, Wei Ding, Michael Giolai, Darren Heavens, Sonja Kersten, Derek S. Lundberg, Manuela Neumann, Julian Regalado, Richard A. Neher, Eric Kemen, and Detlef Weigel

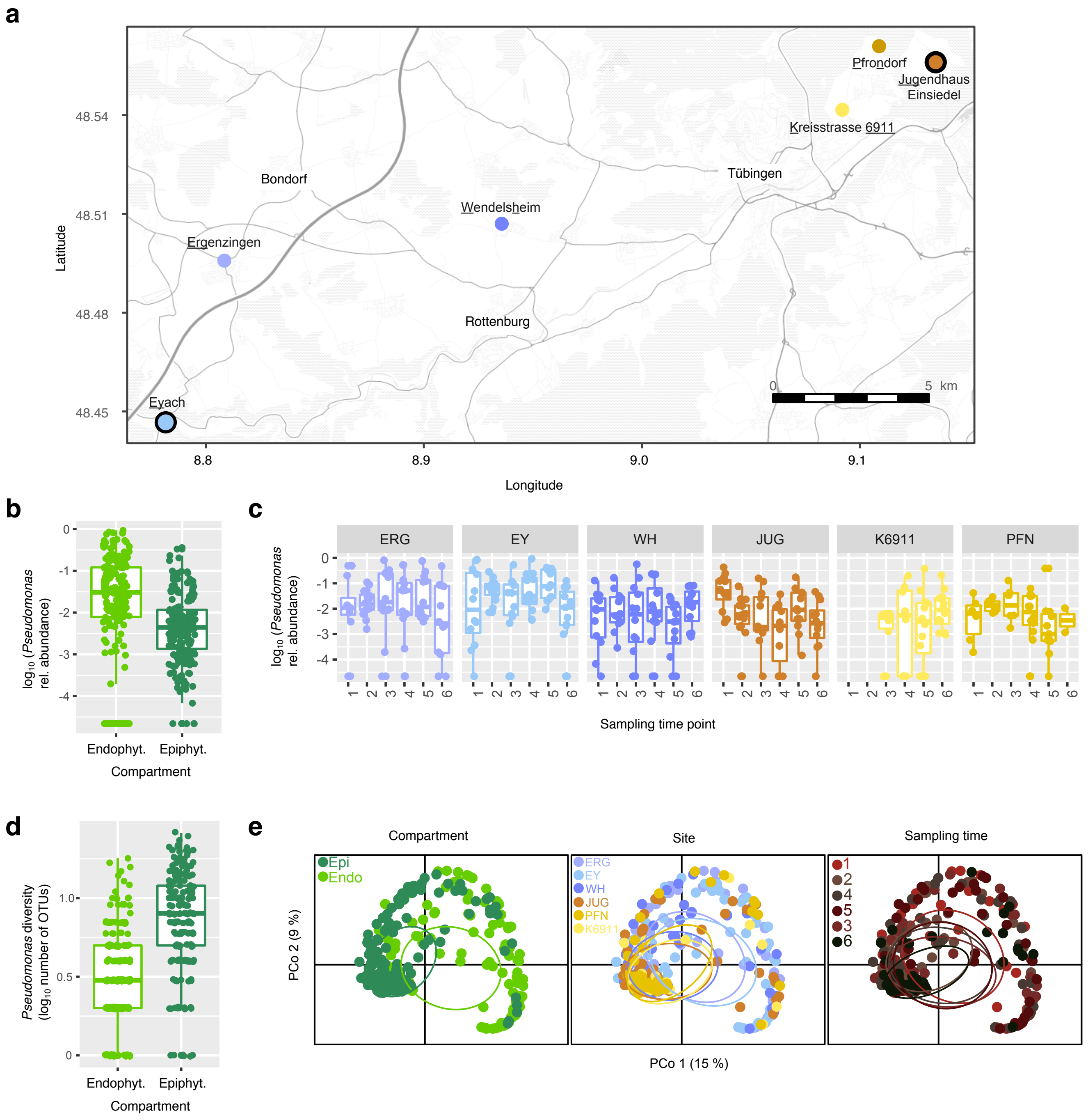

Fig. S1. Changes in *Pseudomonas* populations colonizing *A. thaliana* leaves. Related to Fig. 1. (a) Location of the sampling sites around Tübingen (Germany). The two sites from which isolates were cultured indicated by black outlines. (b) *Pseudomonas* abundance in the endophytic (Endo.) and epiphytic (Epi.) compartments. (c) *Pseudomonas* abundance across the different sites and sampling time points (see Fig. 1a). (d) *Pseudomonas* diversity in the endophytic (Endo.) and epiphytic (Epi.) compartments. (e) Principal coordinates analysis (PCoA) based on Bray-Curtis distances, depicting the differences between *Pseudomonas* populations across the different compartments, sites and sampling time points. *Pseudomonas* relative abundance (RA) was calculated as the ratio of *Pseudomonas* reads to the total number of bacterial reads.

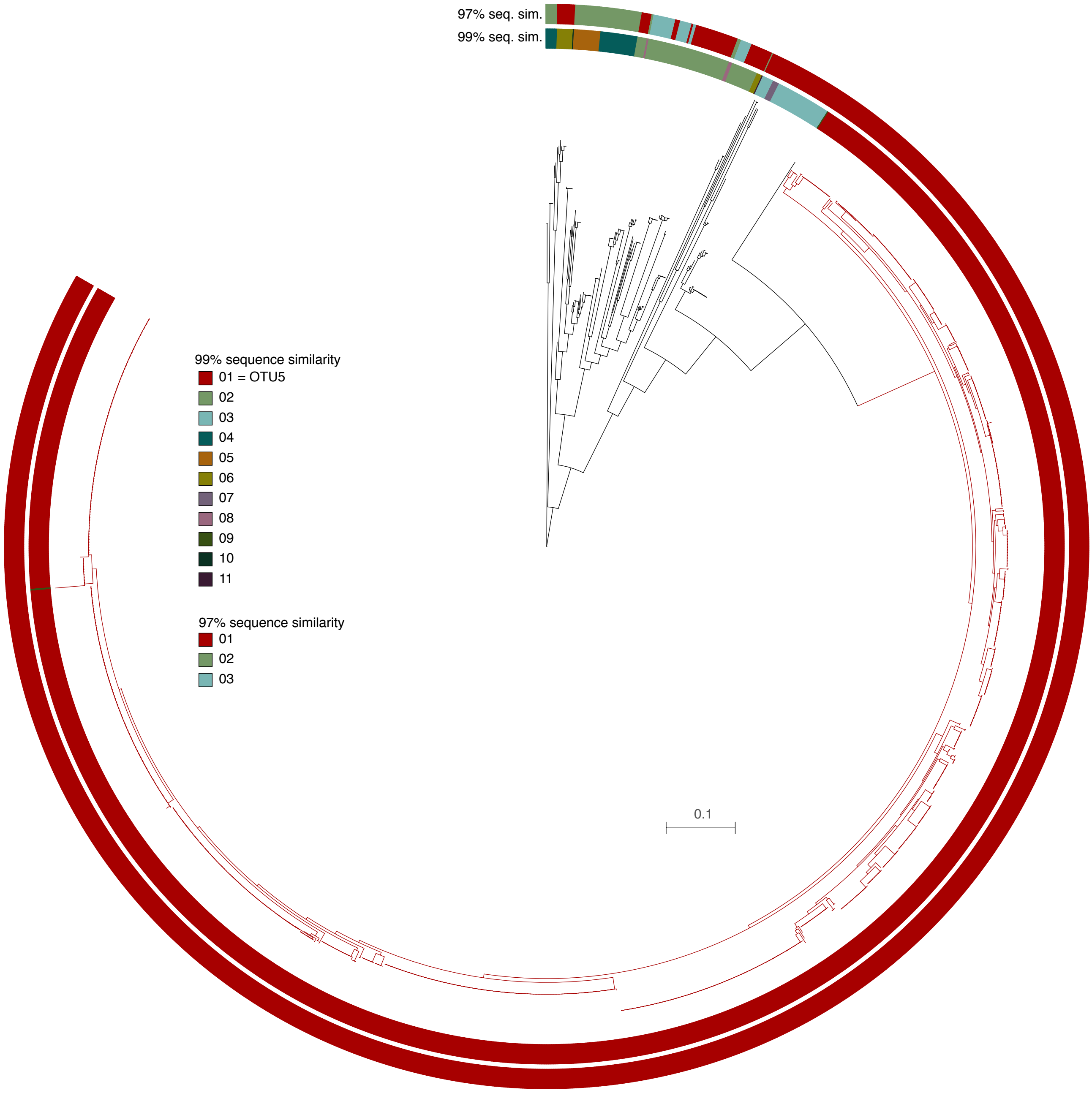

Fig. S2. Pseudomonas OTUs and core-genome phylogeny. Related to Fig. 1, 2 and 3.16S rDNA sequences were extracted from 1,524 Pseudomonas isolates with core-genome sequences. Clustering based on 99% or 97% sequence identity of the v3-v4 region of the 16S rDNA is compared to an ML core genome phylogenetic tree. Colors indicate group, ranked by abundance. The most abundant group corresponds to OTU5 (Bordeaux color), with clustering at 99% sequence identity being more consistent with the core genome tree than 97% clustering.

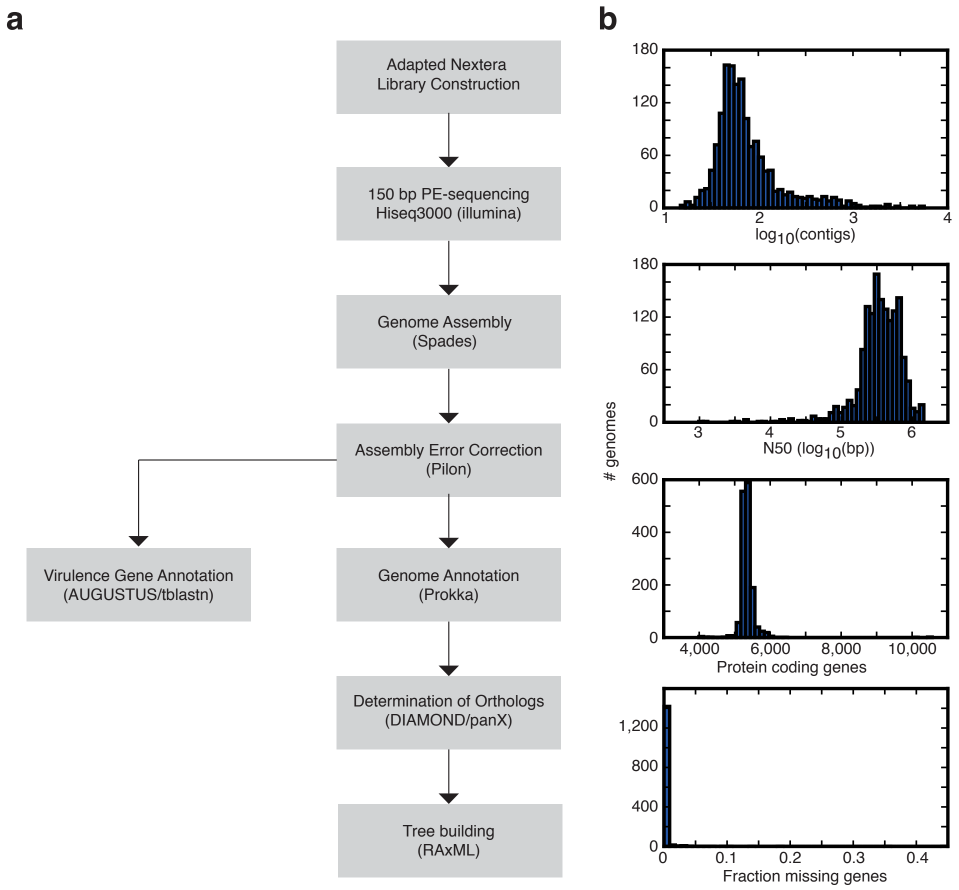

Fig. S3. De novo genome assembly of 1,524 strains sequenced in this study. Related to Fig. 4. Genomes were filtered for basic assembly quality (N50>25,000 bp, and >3,500 genes per genome). (a) The number of contigs and (b) N50, (c) the number of protein coding genes annotated, and (d) the percentage of genes predicted to be missing for each assembly are shown for the 1,524 genomes remaining after filtering.

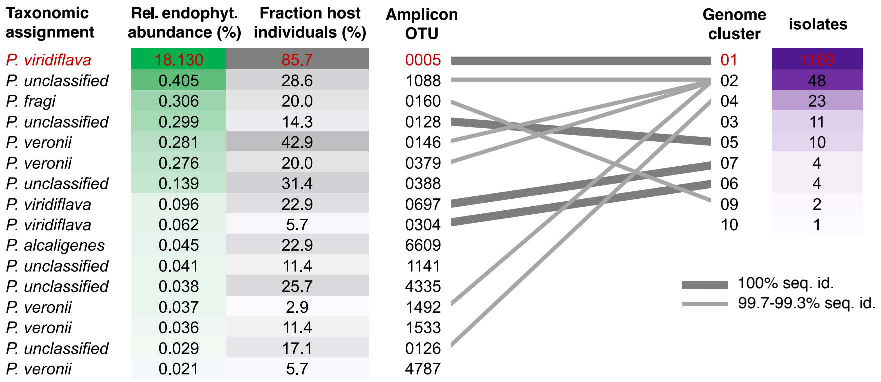

Fig. S4. Leaf-endophytic Pseudomonas diversity at Eyach site: overlap between amplicon sequencing and strain isolation data. Related to Fig. 5. Memberships in strain clusters identified by 99% sequence identity of the 16S rDNA v3-v4, either through amplicon sequencing (OTUs, left) or strain isolation (genomes, right). Matching groups (grey lines) were identified by comparing representative sequences for the amplicon OTUs/genome clusters. Even when considering data from different host individuals sampled at different times there is a good overlap between the two collections.

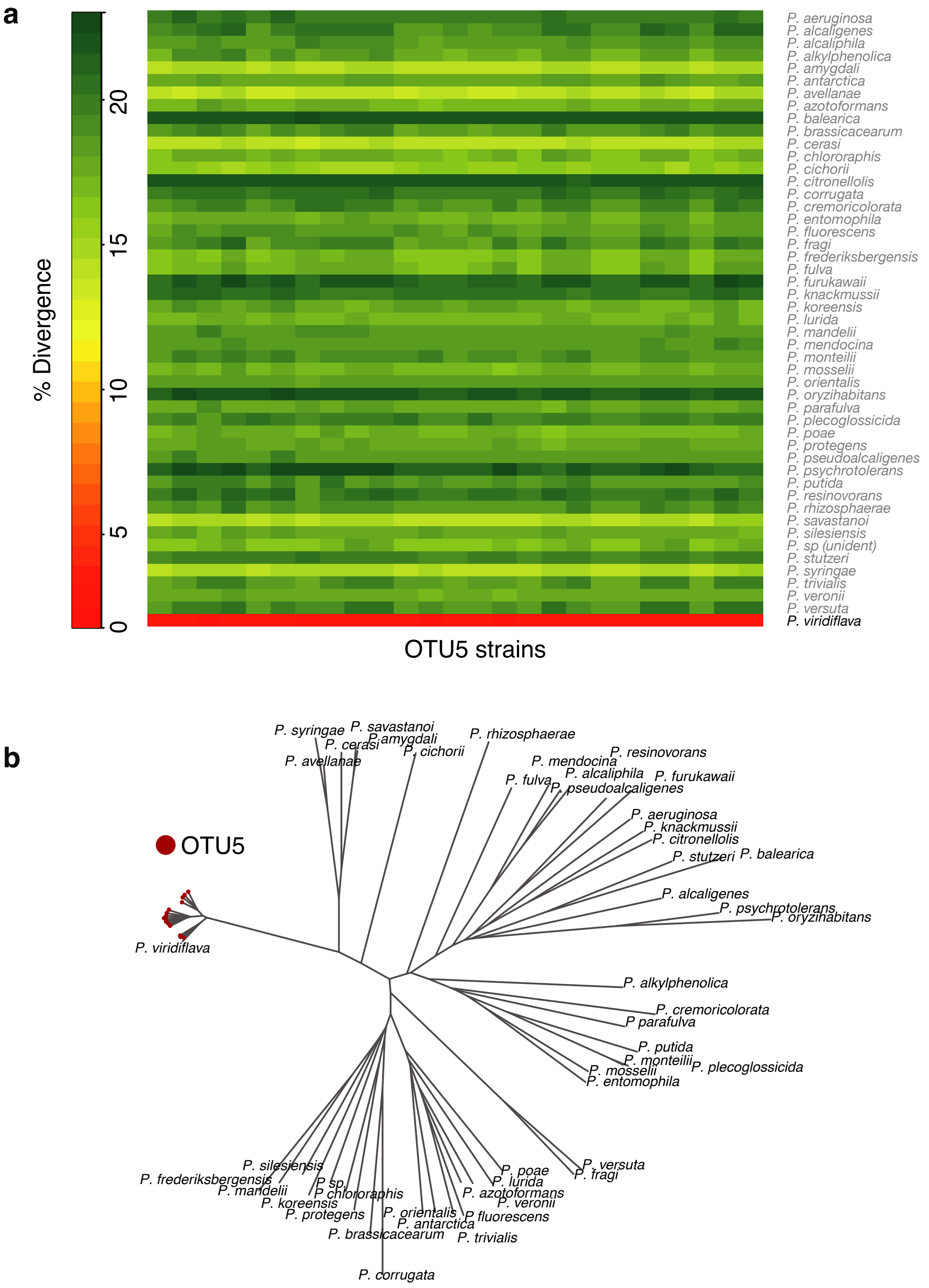

Fig. S5. Pairwise divergence between OTU5 and reference *Pseudomonas* genomes. Related to Fig. 1f. (a) Pairwise divergences between 25 randomly selected OTU5 genomes and a single representative reference genome from each of 51 *Pseudomonas* species were calculated using mash (Ondov et al., 2016). OTU5 strains share the greatest sequence similarity with *P. viridiflava*, differing on average by 2.6%. (b) A Neighbour-joining tree (Saitou and Nei, 1987) was constructed from the pairwise distances, again demonstrating the relatedness of OTU5 to *P. viridiflava*.

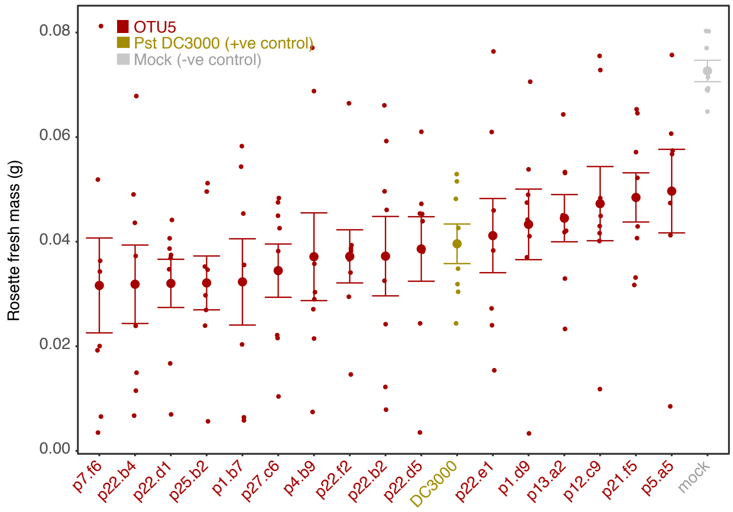

Fig. S6. Gnotobiotic trial with OTU5 strains. Related to Fig. 3c. 14-day old *A. thaliana* plants of accession Eyach 15-2 were drip-infected with different OTU5 strains. All tested strains significantly reduced plant growth (n=8 replicates per sample, Student's t-test, q-value <0.05).

Table S1. Collection locations and dates for all samples. Related to Fig. 1

Site	Latitude	Longitude	Sample type
EY	48.446111	8.781611	16S, metagenome, isolates
K6911	48.541278	9.0925	16S, metagenome
PFN	48.561087	9.109294	16S, metagenome
JUG	48.555722/48.556255	9.134833/9.135424	16S, metagenome, isolates
WH	48.506827	8.936418	16S
ERG	48.495362	8.809083	16S

Table S2. Genes annotated in plant-associated pathways and amino acid sequence of flg22 variants in OTU5. Related to Fig. 6. Top: The listed genes (right column) were custom-annotated in each of the genomes to ascertain the presence of the listed pathway (left column). Bottom: Left column lists the genome from which flg22 sequence was extracted. OTU5 encodes two major variants of flg22, one similar to a *P. floridae* genotype, and the other to a *P. viridiflava* genotype.

Pathway	Components
Coronatine	*cfa1, cfa2, cfa3, cfa4, cfa5, cfa6, cfa7, cfa8, cfa9, cmaA, cmaB, cmaC, cmaD, cmaE, cmaT*
Mangotoxin	*mboA, mboB, mboC, mboD, mboE, mboF*
Phaseolotoxin	*argK, amtA*
Syringomycin	*syrB1, syrB2, syrC, syrD, syrE, syrP*
Syringopeptin	*sypA, sypB, sypC*
Tabtoxin	*tblA, tabA, tabB*

Reference	flg22 Sequence
Pst DC3000	QRLSTGSRINSAKDDAAGLQIA
P. floridae	
(WP_083186033)	ERLSTGKKINTAADDAGGSITQ
OTU5 (649 isolates)	ERLSTGKKINTAADDAGGSITQ
P. viridiflava	
(WP_025995199)	SRLSSGLKVQNARDNVGVLSTI
OTU5 (670 isolates)	SRLSSGLKVQNARDNVGVLSTI
OTU5 (22 isolates)	SRLSSGLKVTNARDNVGVLSTI
Marinomonas sp.	QRLSSGKRINSAKDDAAGMQI
OTU5 (3 isolates)	QKLSSGKSITSSKDNAAGSQIA
