# Supplementary material for: Arabidopsis thaliana and Pseudomonas Pathogens Exhibit Stable Associations over Evolutionary Timescales
Source: Cell Host Microbe. 2018 Jul 11;24(1):168–179.e4. doi: 10.1016/j.chom.2018.06.011 (PMC6054916; doi:10.1016/j.chom.2018.06.011)
Supplement: Document S2. Article plus Supplemental Information [file mmc2.pdf]

Cell Host & Microbe

Arabidopsis thaliana and *Pseudomonas* Pathogens Exhibit Stable Associations over Evolutionary Timescales

Graphical Abstract

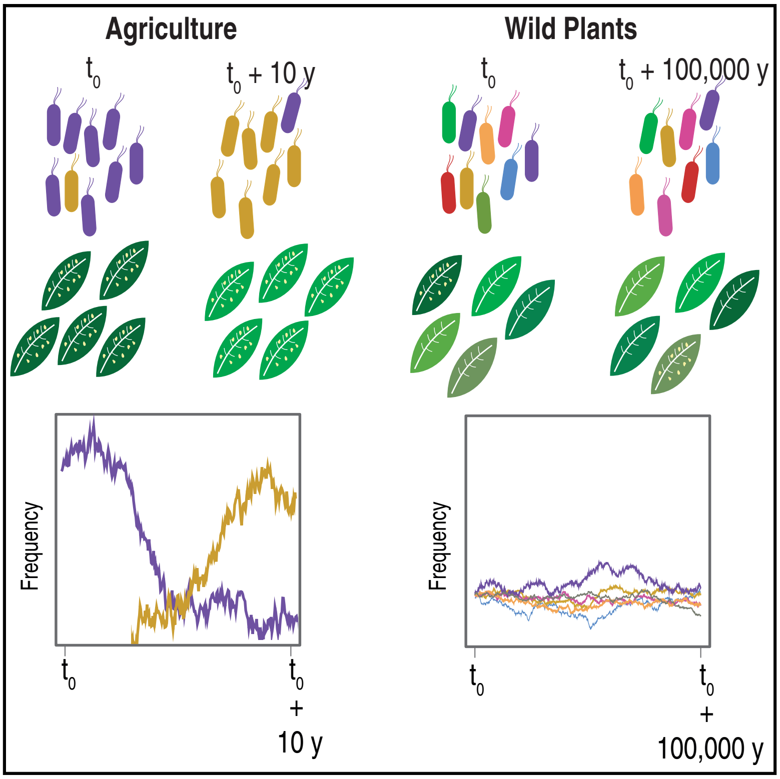

Authors

Talia L. Karasov, Juliana Almario, Claudia Friedemann, ..., Richard A. Neher, Eric Kemen, Detlef Weigel

Correspondence

weigel@tue.mpg.de

In Brief

Disease outbreaks in agriculture are often associated with clonal expansions of single pathogenic lineages. In this study, Karasov et al. show that in populations of a wild plant, no single lineage of an abundant pathogen takes over the host population. Genetic and species diversity may prevent clonal expansions in nature.

Highlights

- Wild *A. thaliana* is regularly colonized by a single *Pseudomonas* OTU
- Strains within this OTU diverged from one another at least 300,000 years ago
- Many strains can cause disease and are classified as pathogenic
- In contrast to agriculture, no single pathogenic strain dominates host populations

Karasov et al., 2018, Cell Host & Microbe *24*, 168–179
July 11, 2018 © 2018 The Author(s). Published by Elsevier Inc.
https://doi.org/10.1016/j.chom.2018.06.011

Arabidopsis thaliana and *Pseudomonas* Pathogens Exhibit Stable Associations over Evolutionary Timescales

Talia L. Karasov,[1] Juliana Almario,[2,3,6] Claudia Friedemann,[1,6] Wei Ding,[1] Michael Giolai,[1,4] Darren Heavens,[4] Sonja Kersten,[1] Derek S. Lundberg,[1] Manuela Neumann,[1] Julian Regalado,[1] Richard A. Neher,[5] Eric Kemen,[2,3] and Detlef Weigel[1,7,*]
[1]Department of Molecular Biology, Max Planck Institute for Developmental Biology, 72076 Tübingen, Germany
[2]Max Planck Research Group Fungal Biodiversity, Max Planck Institute for Plant Breeding Research, Carl-von-Linné Weg 10, 50829 Cologne, Germany
[3]Interfaculty Institute of Microbiology and Infection Medicine Tübingen, IMITP, University of Tübingen, 72076 Tübingen, Germany
[4]Earlham Institute, Norwich Research Park Innovation Centre, Colney Lane, Norwich NR4 7UZ, UK
[5]University of Basel, Klingelbergstrasse 50/70, 4056 Basel, Switzerland
[6]These authors contributed equally
[7]Lead Contact
*Correspondence: weigel@tue.mpg.de
https://doi.org/10.1016/j.chom.2018.06.011

SUMMARY

Crop disease outbreaks are often associated with clonal expansions of single pathogenic lineages. To determine whether similar boom-and-bust scenarios hold for wild pathosystems, we carried out a multi-year, multi-site survey of *Pseudomonas* in its natural host *Arabidopsis thaliana*. The most common *Pseudomonas* lineage corresponded to a ubiquitous pathogenic clade. Sequencing of 1,524 genomes revealed this lineage to have diversified approximately 300,000 years ago, containing dozens of genetically identifiable pathogenic sublineages. There is differentiation at the level of both gene content and disease phenotype, although the differentiation may not provide fitness advantages to specific sublineages. The coexistence of sublineages indicates that in contrast to crop systems, no single strain has been able to overtake the studied *A. thaliana* populations in the recent past. Our results suggest that selective pressures acting on a plant pathogen in wild hosts are likely to be much more complex than those in agricultural systems.

INTRODUCTION

In agricultural and clinical settings, pathogenic colonizations are frequently associated with expansions of a small number of genetically identical microbial lineages (Butler et al., 2013; Cai et al., 2011; Kolmer, 2005; Park et al., 2015; Yoshida et al., 2013). While such epidemics are favored by low genetic diversity of the host (Zhu et al., 2000) and absence of competing microbes (Brown et al., 2013), many, if not most, pathogens can colonize host populations that are both genetically diverse and can accommodate a diversity of other microbes (Falkinham et al., 2015; Woolhouse et al., 2001).

Factors that drive pathogen success in such more complex situations are less well understood than for clonal epidemics. For example, if a pathogen persists at high numbers in non-host environments, does each host become infected by a different pathogen strain? Or is each host infected by a multitude of genetically distinct strains? And do different colonizing strains use the same mechanisms to overcome host defenses? The answers to these questions inform on how (and if) a host population can evolve partial or even complete pathogen resistance (Anderson and May, 1982; Barrett et al., 2009; Karasov et al., 2014a; Laine et al., 2011). Several studies over the past 20 years have attempted to infer the distributions of non-epidemic pathogens in both host and non-host environments (Falkinham et al., 2015; Wiehlmann et al., 2007). These studies have observed a range of different patterns, with varying conclusions for different collections, even of the same pathogen species (Pirnay et al., 2009). What has become clear is that non-epidemic pathogens are phenotypically polymorphic, but the underlying scale and pattern of genetic and genomic differentiation remain unknown (Kniskern et al., 2011; Thrall et al., 2001).

Questions of pathogen epidemiology are of particular relevance when considering the genus *Pseudomonas*, which includes pathogens and commensals of both animals and plants and is among the most abundant genera in plant leaf tissue (Jakob et al., 2002). Of the well over 100 recognized *Pseudomonas* species belonging to the Gram-negative gammaproteobacteria (Gomila et al., 2015), three of the most commonly found on plants are *P. syringae* and *P. viridiflava* in the *P. syringae* complex and *P. fluorescens* (Bartoli et al., 2014). *Pseudomonas* can have a large impact on plant fitness (Balestra et al., 2009; Yunis et al., 1980), and several putatively host-adapted lineages, which are distinguished by the repertoire of disease-causing genes, can trigger agricultural disease epidemics (Baltrus et al., 2011, 2012). But despite the damage they can do to plants, *Pseudomonas* pathogens are not obligatory biotrophs: surveys in environmental and non-host habitats have revealed distribution

 © 2018 The Author(s). Published by Elsevier Inc.
This is an open access article under the CC BY license (http://creativecommons.org/licenses/by/4.0/).

patterns typical for opportunistic microbes (Morris et al., 2010), with genetically divergent lineages not uncommonly found in the same host population (Barrett et al., 2011; Karasov et al., 2017).

To understand how the distribution of a common plant pathogen differs between agricultural and non-agricultural situations, we have begun to elucidate the epidemiology of *Pseudomonas* strains within and between populations of a non-agricultural host. *Arabidopsis thaliana* is a globally distributed wild plant capable of colonizing poor substrates as well as fertilized soils (Weigel, 2012). *Pseudomonas* is commonly found on and in *A. thaliana* leaves, and many of these strains can cause disease, even though they are likely not specialized on *A. thaliana* as a host (Barrett et al., 2011; Jakob et al., 2002; Kniskern et al., 2011).

Here we report a broad-scale survey of *Pseudomonas* operational taxonomical units (OTUs) based on 16S rDNA sequences in six *A. thaliana* populations from Southwestern Germany, over six seasons. A single OTU was found to consistently dominate in individual plants, across populations, and across seasons. Through subsequent sequencing of 1,524 *Pseudomonas* genomes, we uncovered extensive diversity within this pathogenic OTU, diversity that is much older than *A. thaliana* is in the surveyed area. Taken together, this makes for a colonization pattern that differs substantially from what is typically observed for crop pathogens. The observation of a single dominant and temporally persistent *Pseudomonas* lineage in several host populations is at first glance reminiscent of successful pathogens in agricultural systems. However, in stark contrast to many crop pathogens, this *Pseudomonas* pathogen can apparently persist as a diverse metapopulation over long periods, without a single sublineage becoming dominant.

RESULTS

Dozens of *Pseudomonas* OTUs Persist in *A. thaliana* Populations

To obtain a first understanding of local diversity of *Pseudomonas*, which is abundant in *A. thaliana* populations from Southwestern Germany (Agler et al., 2016), we analyzed the v3-v4 region of 16S rDNA sequences from epi- and endophytic leaf compartments, across six host populations in spring and fall of three consecutive years (Figures 1A and S1A; Table S1). *Pseudomonas* was found in 97% of epi- and 88% of endophytic samples, representing 2% and 10% of the total bacterial community in each compartment, respectively. Densities were higher in the endophytic compartment (ANOVA, R^2 = 6.8%, $p = 10^{-7}$; Figure S1B), indicating a preferential colonization of this niche. While we did not detect an effect of sampling time (ANOVA, $p > 0.05$), the relative abundance of *Pseudomonas* varied also across sites (ANOVA, R^2 = 7.9%, $p = 10^{-6}$; Figure S1C).

By clustering *Pseudomonas* 16S rDNA reads at 99% sequence identity, we could distinguish 56 OTUs (Figure 1B). The 99% threshold resulted in OTU patterns more congruent with a subsequently derived core genome-phylogeny than the more widely used 97% sequence identity (Figure S2). Thirteen of the 56 OTUs, including the most abundant OTU, OTU5, were classified as *P. viridiflava*, which belongs to the *P. syringae* complex. The other classifiable OTUs belonged to the *P. fluorescens*, *P. aeruginosa*, and *P. stutzeri* species complexes (Figure 1B).

To understand the factors shaping *Pseudomonas* assemblages, we studied variation in OTU presence and relative abundances as an indication of population structure. Permutational multivariate ANOVA (PerMANOVA) on Bray-Curtis distances indicated that differences between host individuals were associated primarily with interactions between site, leaf niche, and sampling time (20% explained variance; $p < 0.05$), with a smaller percentage associated with each factor independently such as site (4% explained variance), leaf niche (5%), or sampling time (3%). An important difference between leaf niches was that endophytic *Pseudomonas* populations were almost three times less diverse than epiphytic populations (Wilcoxon test, $p < 10^{-16}$) (Figure S1D), pointing to stronger selection inside the leaf.

A Single Lineage Dominates *Pseudomonas* Populations in *A. thaliana* Leaves

OTU5 was overall the most common *Pseudomonas* OTU across samples (Figure 1B), occurring in 59% of epi- and 58% of endophytic samples. Across all samples, OTU5 accounted for almost half of all reads identified as *Pseudomonas* in the endophytic compartment (48%, range 0%–99.9% in each sample), and it was the most abundant endophytic *Pseudomonas* OTU in 52% of samples. The dominance of OTU5 was less pronounced in the epiphytic samples, where it averaged 23% of all reads (range 0%–99.9%), being the top OTU in only 22% of samples (Figures 1C and 1D), indicating an enrichment in the endophytic compartment (Wilcoxon test, $p = 1.0 \times 10^{-4}$; paired Wilcoxon test, $p = 1.0 \times 10^{-15}$; Figure 1E). In conjunction with the overall reduced *Pseudomonas* diversity in this compartment, this is evidence for OTU5 strains being particularly successful endophytic colonizers of *A. thaliana*.

16S rDNA reads reveal the relative abundance of microbes, but they do not inform on the absolute abundance of microbial cells in a plant, what we term the "microbial load." 16S rDNA analysis might indicate that a pathogen dominates the microbiota, but unless it reaches a certain absolute level, there might not be a marked decrease in host fitness (Duchmann et al., 1995; Schneider and Ayres, 2008; Vaughn et al., 2000). The importance of absolute microbial load has recently come into focus of human gut microbiome analyses as well (Vandeputte et al., 2017).

To determine whether OTU5 abundance in individual samples reflected excessive OTU5 growth, or merely successful suppression of other microbes, we quantified total microbial colonization by estimating the ratio of microbial over plant host reads in metagenome shotgun sequencing data. We returned to four of the previously sampled populations (Figures 1A and S1A); collected and extracted genomic DNA from entire, washed leaf rosettes; and whole-genome shotgun sequenced 176 plants. The same material was used to call OTUs from 16S rDNA v4 region amplicons.

We mapped Illumina reads against all bacterial genomes in GenBank and against the *A. thaliana* reference genome, and determined the ratio of bacterial to plant reads. Microbial load varied substantially across the 176 plants (Figure 2A), and we calculated its correlation with each of the 3,647 OTUs detected in at least one sample. Because OTUs were called on 16S

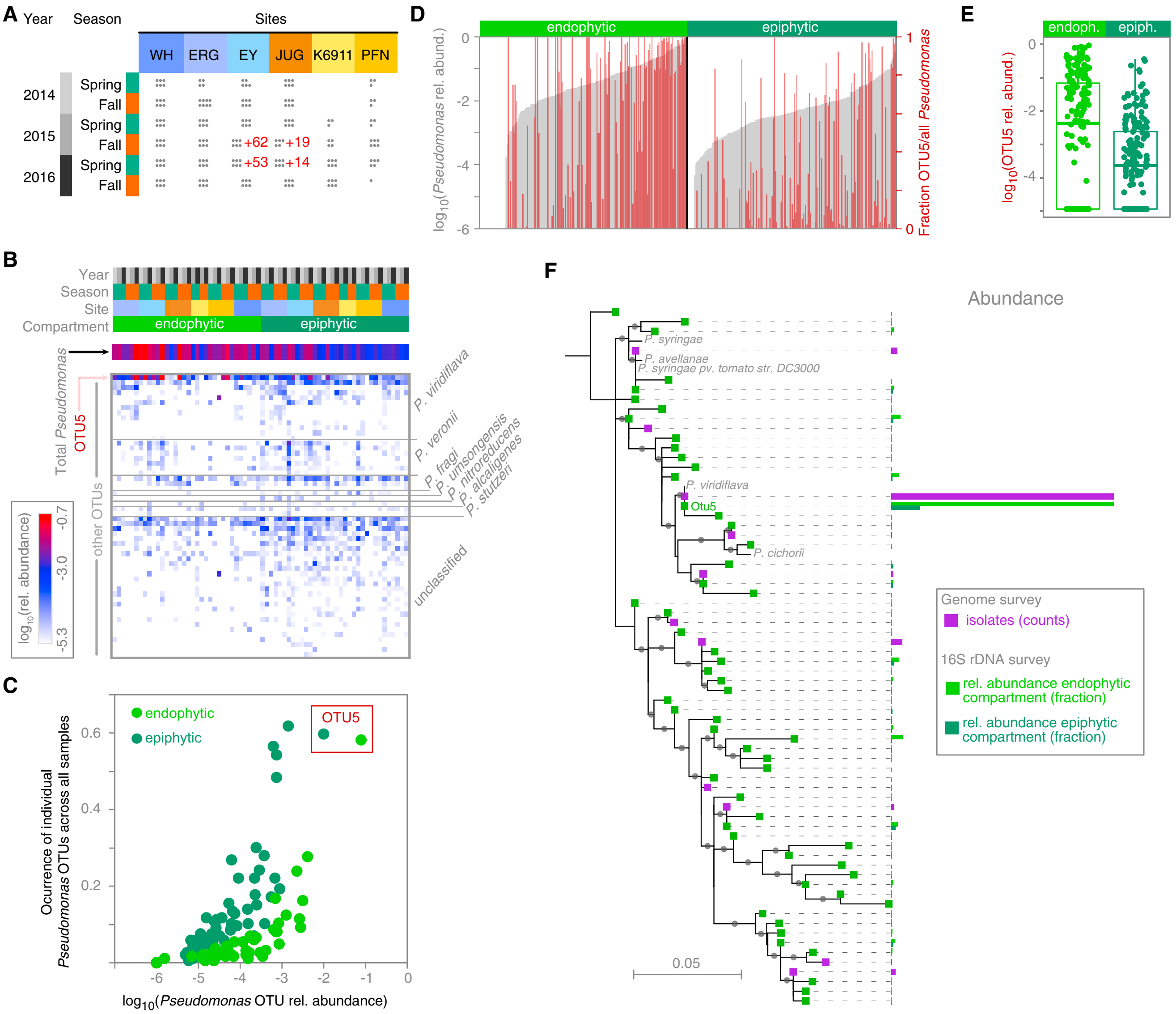

Figure 1. Natural *Pseudomonas* Populations in *A. thaliana* Leaves Are Dominated by the OTU5 Lineage

(A) Overview of 16S rDNA survey of epi- and endophytic compartments of *A. thaliana* plants (dots indicate sampled plants). Red numbers indicate individuals from which *Pseudomonas* isolates were cultured and metagenome analysis was performed in parallel.

(B) Heatmap of relative abundance of 56 *Pseudomonas* OTUs in the 16S rDNA survey. Color key to samples on top according to (A). *Pseudomonas* species assignments on the right. *P. veronii*, *P. fragi*, and *P. umsongensis* belong to the *P. fluorescence* complex; *P. nitroreducens* and *P. alcaligenes* to the *P. aeruginosa* complex.

(C) Correlation between occurrence across all samples and average relative abundance within samples of the 56 *Pseudomonas* OTUs in the endo- and epiphytic compartments.

(D) *Pseudomonas* abundance (gray bars) and percentage of *Pseudomonas* reads belonging to OTU5 (red bars), in the endo- and epiphytic compartments.

(E) OTU5 is significantly more abundant in the endophytic compartment (Wilcoxon test, $p = 10^{-4}$).

(F) ML phylogenetic tree illustrating the similarity between amplicon sequencing-derived and isolation-derived *Pseudomonas* OTUs defined by distance clustering at 99% sequence identity of the v3-v4 regions of the 16S rDNA. For isolate OTUs, exact 16S rDNA sequences were used; for amplicon sequencing OTUs, the most common representative sequence was used. Gray dots on branches indicate bootstrap values >0.7. Colored bars represent the relative abundance or the number of isolates. The most abundant *Pseudomonas* OTU in both the endophytic and epiphytic compartments, OTU5, was identical in sequence to the most abundant sequence observed among isolates and to a *P. viridiflava* reference genome (NCBI AY597278.1/AY597280.1).

See also Figures S1, S2, and S5.

rDNA amplicons, but microbial load was assessed on metagenomic reads, the two assays provided independent measurements of relative and absolute microbe abundance. Among all OTUs, a sequence that matched with OTU5 at 100% sequence identity over the v4 region was the most positively correlated with total microbial load (Figures 2B and 2C; Pearson correlation coefficient R = 0.41, q value = 6×10^{-6}), indicating not only that the OTU5 strains are the most common *Pseudomonas* strains in

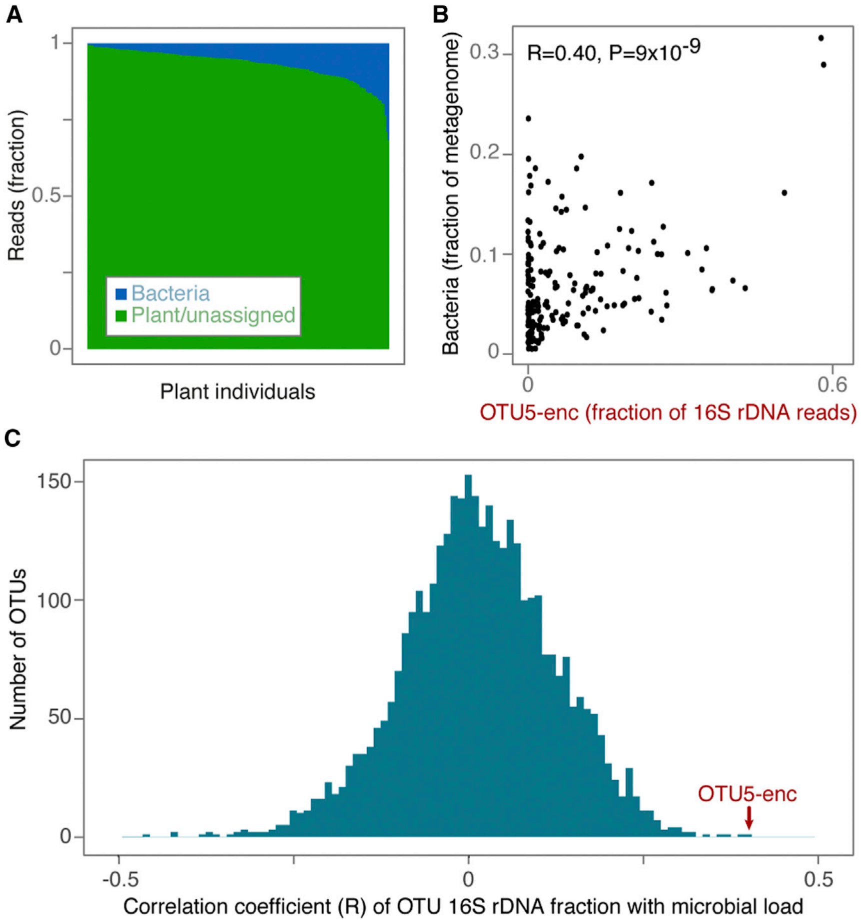

Figure 2. The Most Abundant OTU, which Encompasses OTU5 (OTU5-enc), Is Correlated with Microbial Load

(A) Bacterial and plant fraction of metagenome shotgun sequencing reads in 176 plants.

(B) Correlation between fraction of bacterial reads in metagenome data and relative abundance of OTU with 100% identity to OTU5 in the v4 region of 16S rDNA amplicons from the same 176 samples.

(C) Distribution of Pearson correlation coefficients between microbial loads as inferred from fraction of bacterial reads and OTU abundances (as shown for OTU5 in B). The correlation coefficient for the OTU5-associated sequence abundance is the highest among any of the 3,647 OTUs detected across all samples.

See also Figure S2.

these plants, but also that they are either major drivers or beneficiaries of microbial infection in these plants.

OTU5 Comprises Many Genetically Distinct Strains

Because a single 16S rDNA OTU can include genetically and phenotypically diverse strains (Moeller et al., 2016), we set out to compare the complete genomes of OTU5 strains. From the same plants in which we had analyzed the metagenomes, we cultured between 1 and 34 *Pseudomonas* colonies (mean = 11 per plant, median = 12). We then assembled *de novo* the full genomes of 1,611 *Pseudomonas* isolates selected without any prior OTU assignment (assembly statistics in Figure S3). Eighty-seven genomes with poor coverage, abnormal assembly characteristics, or incoherent genome-wide sequence divergence were removed from further analysis. The remaining 1,524 genomes were 99.5% complete based on standard criteria (Simão et al., 2015), containing on average 5,347 predicted genes (SD 284). Extraction of 16S rDNA sequences demonstrated that the vast majority of all isolates, 1,355, belonged to the OTU5 lineage, as defined previously by amplicon sequencing (Figure S4).

Maximum-likelihood (ML) core genome phylogenies (Ding et al., 2018) were constructed from the concatenation of 939 genes that classified as the aligned soft core genome of our *Pseudomonas* collection. Because bacteria undergo homologous recombination, the branch lengths of the ML core genome tree may not properly reflect the branch lengths of vertically inherited genes, but the overall topology is expected to remain consistent (Hedge and Wilson, 2014). The 1,524-genome phylogeny revealed hundreds of isolates with a core genome that was nearly or completely identical to that of at least one other isolate. Using a 99.9% sequence identity cutoff (corresponding to a SNP approximately every 1,000 bp across the core genome based on distance in the ML tree), our isolates collapsed into 165 distinct strains (Figure 3A). In the core genome tree, 1,355 OTU5 isolates, comprising 82 distinct strains, formed a single monophyletic clade. One genome (p8.A2) in this clade that differed in its 16S rDNA taxonomical assignment apparently represented a mixture of two isolates. In support of the 16S rDNA placement of OTU5 within the *Pseudomonas* genus, the OTU5 clade is most closely related to *P. viridiflava* and *P. syringae* strains (Figure 1F). The NCBI (April 2018) reference genome most similar to OTU5 is classified as *P. viridiflava* (GenBank: GCA_900184295.1), with an average of 2.6% divergence (Figure S5). Genetic differences between our strains were distributed throughout the genome, indicating that divergence was not solely the result of a few importation events of divergent, horizontally transferred material (Figure 4A).

Comparing the position of strains on the phylogeny and their provenance identified several strains that were not only frequent colonizers across plants, but also persistent colonizers over time, each isolated in at least two consecutive seasons (Figure 5A). Five OTU5 strains accounted for 46% of sequenced isolates, each with an overall frequency of between 4% and 10%, with several found in over 20% of plants. In contrast, no strain outside OTU5 exceeded an overall frequency of 5%. Generally, non-OTU5 strains were much less likely to be represented by multiple isolates and were rarely observed in both seasons sampled.

OTU5 Comprises Many Potentially Pathogenic Strains, but with Distinct Phenotypes

The *P. syringae/viridiflava* complex, to which OTU5 belongs, contains many well-known plant pathogens—although not all

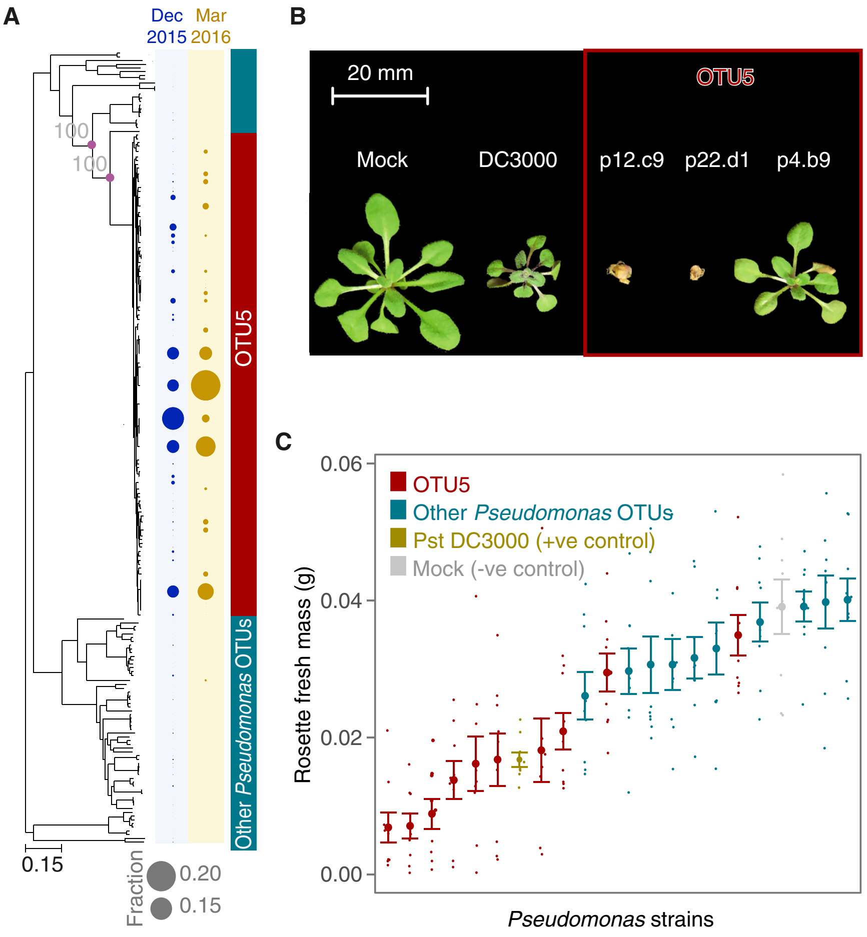

Figure 3. OTU5 Is Composed of Multiple Expanding Lineages that Are Pathogenic

(A) ML whole-genome phylogeny and abundance of strains in Eyach, Germany, in December 2015 and March 2016. Diameters of circles on the right indicate relative abundance across all isolates from that season. Purple circles at nodes relevant for OTU5 classification and gray numbers indicate support with 100 bootstrap trials.

(B) Examples of OTU5 strains that can reduce growth and even cause obvious disease symptoms in gnotobiotic hosts.

(C) Quantification of effect of drip infection on growth of plants. Pst DC3000 was used as positive control. The negative control did not contain bacteria.

Values represent mean ± SEM. See also Figures S2 and S5.

strains in this complex are pathogenic, with some lacking the canonical machinery required for virulence (Clarke et al., 2010). Because some infection characteristics are determined by the presence of a single or few genes, even closely related strains can cause diverse types of disease (Kniskern et al., 2011). Given the known phenotypic variability within and between *Pseudomonas* species, 16S rDNA sequences alone did not inform on the pathogenic potential of the OTU5 strains.

To directly determine the pathogenicity—which we define here as the ability to cause disease in the laboratory—of diverse OTU5 isolates, we drip-inoculated 26 of them on seedlings of Eyach 15-2, an *A. thaliana* genotype common at one of our sampling sites (Bomblies et al., 2010) (Figures 3B, 3C, 4A, and S6). All but one of these reduced plant growth significantly; this was the case for only two of ten randomly chosen non-OTU5 *Pseudomonas* strains (ANOVA, $p < 0.05$). The tomato pathogen *P. syringae* pv. tomato (Pst) DC3000, which is highly pathogenic on *A. thaliana* (Velásquez et al., 2017), reduced growth to a similar extent as several OTU5 strains, with some OTU5 strains being even more pathogenic (Figures 3B and 3C). Treatment of seedlings with boiled, dead bacteria from five different OTU5 isolates did not reduce plant growth (ANOVA, $p > 0.25$ for all), indicating that disease symptoms were not due to run-away immunity triggered by the initial inoculation, but were indeed caused by proliferation of living bacteria. From the clear phenotypic stratification of strains, we conclude that most OTU5 strains are pathogenic. These results, in conjunction with the observed correlation of OTU5 with microbial load in the field, established with metagenomic methods, point to OTU5 as being responsible for some of the most persistent bacterial pathogen pressures in the sampled *A. thaliana* populations.

Strains within OTU5 Diverged over 300,000 Years Ago

Crop pathogen epidemics are frequently due to few, if not single, strains, with the dominant strains often changing over the course of a few years or decades (Kolmer, 2005; McCann et al., 2017; Yoshida et al., 2013). For example, when Cai and colleagues followed *P. syringae* strains in tomato fields during the 20th century, they found that almost always only one or two strains were present at high frequency (Cai et al., 2011). Isolates from the lineage that was most abundant over the last 60 years—to which today over 90% of assayed isolates belong—differed at only a few dozen SNPs throughout the genome, indicative of a common ancestor as recently as just a few decades ago.

Our OTU5 isolates were much more diverse, with over 10% of positions (27,217/221,628 bp) in the OTU5 core genome being polymorphic. However, the age of diversification cannot be inferred directly from a concatenated whole-genome tree (Hedge and Wilson, 2014) because recombination events with horizontally transferred DNA can increase divergence between strains, thereby elongating branches and inflating estimates of the time to the most recent common ancestor (TMRCA). To prevent TMRCA overestimation, it was necessary to correct for the effects of recombination. This can lead to an underestimate of branch lengths (Hedge and Wilson, 2014), which we found acceptable because we wanted to learn the lower bound for TMRCA among the OTU5 isolates. Inference of recombination sites in the core genome (Didelot and Wilson, 2015) allowed us to remove 7,646 recombination tracts, which

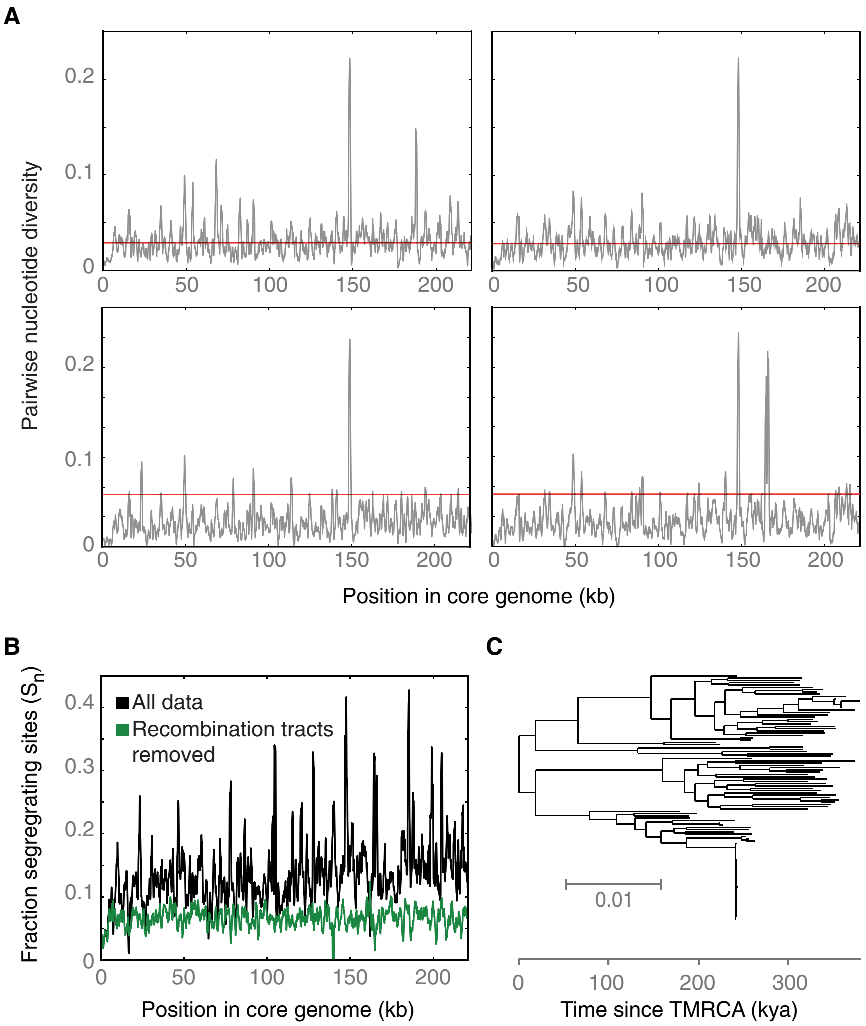

Figure 4. Genome-wide Divergence and Dating of OTU5 Strains

(A) Pairwise nucleotide diversity in 1,000 bp sliding windows. One randomly chosen OTU5 reference strain was separately compared with four different other OTU5 strains.

(B) Genome-wide distribution of segregating sites (S_n) in OTU5, calculated in 1,000 bp sliding windows. Putative recombination tracts were removed from the core genome alignment to calculate the coalescence of OTU5. This removal reduced the fraction of segregating sites by half (0.14 versus 0.07).

(C) The TMRCA of 107 isolates representing the genetic diversity of OTU5 strains as calculated using a substitution rate estimated in McCann et al. (2017).

See also Figure S3.

eliminated about half of all segregating sites and made the distribution of polymorphic sites across the genome more even (Figure 4B).

For neutral coalescence, it is ideal to consider only 4-fold degenerate sites, but because this would have left too few segregating sites, we included all non-recombined sites in our TMRCA calculations. The ML tree of 107 OTU5 isolates that span the diversity of the OTU5 clade with recombination events removed contained a median mid-point-root-to-tip distance of 0.026 (SD = 0.004). McCann and colleagues (McCann et al., 2017) have deduced that a clonal kiwi pathogen lineage related to OTU5 accrues 8.7×10^{-8} substitutions per site per year, and application of this rate led to a TMRCA estimate of 300,000 years (SD of root-to-tip distances = 46,000 years) (Figure 4C). This is likely an underestimate of the TMRCA, due to removal of ancient homoplasies (Hedge and Wilson, 2014). Furthermore, the short-term substitution rate in the kiwi pathogen is likely higher than the long-term substitution rate relevant to OTU5 (Exposito-Alonso et al., 2018; Kryazhimskiy and Plotkin, 2008; Rocha et al., 2006). Nevertheless, we conclude that OTU5 strains likely diverged from one another approximately 300,000 years ago, pre-dating the recolonization of Europe by *A. thaliana* from Southern refugia after the Last Glacial Maximum (1001 Genomes Consortium, 2016).

Individual Pathogenic Strains Often Dominate *In Planta*

Since multiple isolates (1–34, median 12) had been sequenced from most sampled plants, we could assess the frequency of specific strains not only across the entire population, but also within each individual host. Most plants, 73%, were colonized by multiple strains. While similar numbers of distinct OTU5 and non-OTU5 strains were represented in our population-level survey (Figure 3A), non-OTU5 strains tended to be at low frequencies in individual plants. Of all OTUs, only OTU5 strains were likely to partially or completely dominate within a single plant (Figures 5A and 5B).

We measured the Shannon Index H' (Hill, 1973) to compare strain diversity per plant across the two seasons in which we had sampled isolates. While the fall cohort tended to have been colonized by several strains simultaneously, plants in spring were characterized by reduced strain diversity (Figure 5C) (Student's t test, $p = 1.3 \times 10^{-15}$). One possible explanation for this change in strain frequencies is a local spring bloom of OTU5 populations. Plants sampled in spring carried a significantly higher absolute *Pseudomonas* load (Figure 5D) (Student's t test, $p = 10^{-5}$), consistent with spring conditions favoring local OTU5 proliferation.

Gene Content Differentiation of the OTU5 Clade

The abundance of OTU5 as well as its enrichment in the endo- over the epiphytic compartment indicated that this lineage colonizes *A. thaliana* more effectively than do related OTUs. Whether this success is the result of expansion in the plant, or host filtering of colonizers, is unclear, and we were curious what endows OTU5 strains with apparent capacity to outcompete other *Pseudomonas* lineages and to dominate in populations and in

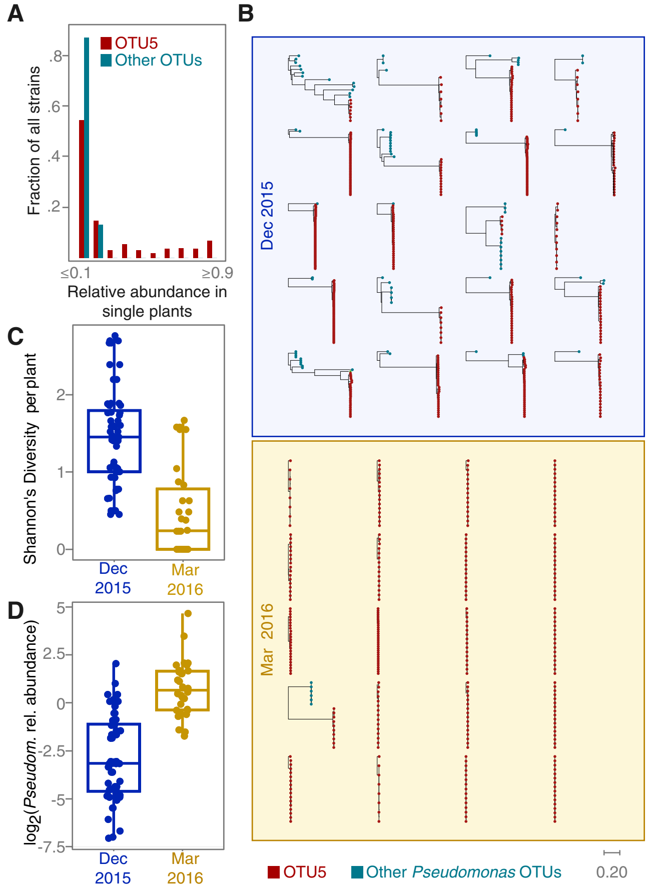

Figure 5. Different OTU5 Strains Expand Clonally within Different Plants

(A) Distribution of relative OTU5 and non-OTU5 strain abundances in single plants.

(B) Phylogenetic trees of isolates collected from individual plants.

(C) Strain diversity as function of season.

(D) *Pseudomonas* load as function of season.

For both (C) and (D), seasons are significantly different (Student's t test, $p = 1.32 \times 10^{-15}$). Boxplots show median and first and third quartiles. Related to Figure S4.

individual plants. To investigate a potential common genetic basis, we assessed the distribution of ortholog groups across all *Pseudomonas* isolates including OTU5 using panX (Ding et al., 2018) (Figures 6A and 6B). From a presence-absence matrix in the pan-genome analysis one can immediately distinguish OTU5 from non-OTU5 lineages. Six hundred and twenty-two genes are conserved (>90% of genomes) within OTU5, but are much more rarely found in other lineages, in fewer than 10% of non-OTU5 strains. A large percentage of the OTU5-specific genes, 25%, encode proteins without known function ("hypothetical proteins").

For successful colonization, microbes often deploy toxins, phytohormones, and effectors that they inject into host cells or the apoplast. To determine whether such compounds are likely to be associated with the abundance of OTU5, we generated a custom database of relevant genes known from *Pseudomonas* and used it to annotate each isolate (Figure 6C; Table S2). OTU5 strains lacked all known genes for coronatine and syringomycin/syringopeptin synthesis, while genes for pectate lyase synthesis were broadly conserved both in and outside of OTU5 (Figure S6). The *hrp-hrc* gene cluster, which encodes the type III secretion system (T3SS) along with effectors and several other proteins involved in pathogenicity (Alfano et al., 2000), is largely conserved across OTU5 isolates, with OTU5 alleles being most similar to the *hrp-hrc* clusters of previously sequenced *P. viridiflava* strains (Figure 6C) (Araki et al., 2006). We note that our search for plant-associated toxins and enzymes is not exhaustive. For example, other plant microbes deploy enzymes that can degrade the cell walls of their hosts (Almario et al., 2017), but such pathways have yet to be identified in *Pseudomonas*.

Plants can detect microbes both through the presence of effector molecules and through microbe-associated molecular patterns (MAMPs). One such well-studied MAMP important in the *Pseudomonas-Arabidopsis* pathosystem is the flg22 peptide in flagellin (Gómez-Gómez et al., 1999). Isolates within OTU5 encode two major flg22 variants, which are highly divergent from one another (Table S2).

Effector proteins increase bacterial fitness in different ways (Chen et al., 2010; Xin et al., 2016) and are thought to be at the forefront of the coevolutionary interaction with the plant immune system (Karasov et al., 2014b). Only one gene for an effector homolog was broadly conserved across OTU5, *avrE*. It was shared with other *P. syringae* type isolates (Dillion et al., 2017), but found rarely outside this group. *avrE* encodes an effector that leads to increased humidity of the extracellular environment inside the plant, the apoplast (Xin et al., 2016). Experimental manipulation of apoplast humidity has shown that it is central to bacterial proliferation within the host. The most abundant *avrE* allele identified in our study is most similar to that previously observed in other *P. viridiflava* strains (Araki et al., 2006), with less similarity to the allele in the well-studied pathogen Pst DC3000 (Figure 6D). The conservation of an *avrE* homolog in members of the OTU5 lineages suggests that it contributes to the success of OTU5 in natural *A. thaliana* populations.

DISCUSSION

In human medicine, an understanding of differences between simplified clinical settings and more complex environments outside the clinic, which are often distinguished by levels of antibiotic treatment, has led to important innovations in the treatment of infections (Bakken et al., 2011). A similar understanding of differences between pathogen colonization and evolution in natural versus agricultural systems may similarly lead to innovations that reduce pathogen pressure in agriculture (Hu et al., 2016). Much can be learned about the course of pathogen colonization and evolution by examining pathogen population diversity and demography. For example, whether pathogen expansions in host populations are composed of single, genetically monomorphic strains or instead comprise numerous genetically divergent strains can indicate whether the successful pathogen lineage has been only recently introduced/evolved or is instead old. Pathogen diversity is not only an indicator of the colonization process, but the diversity itself will also influence the course of colonization and the evolution of resistance in host populations.

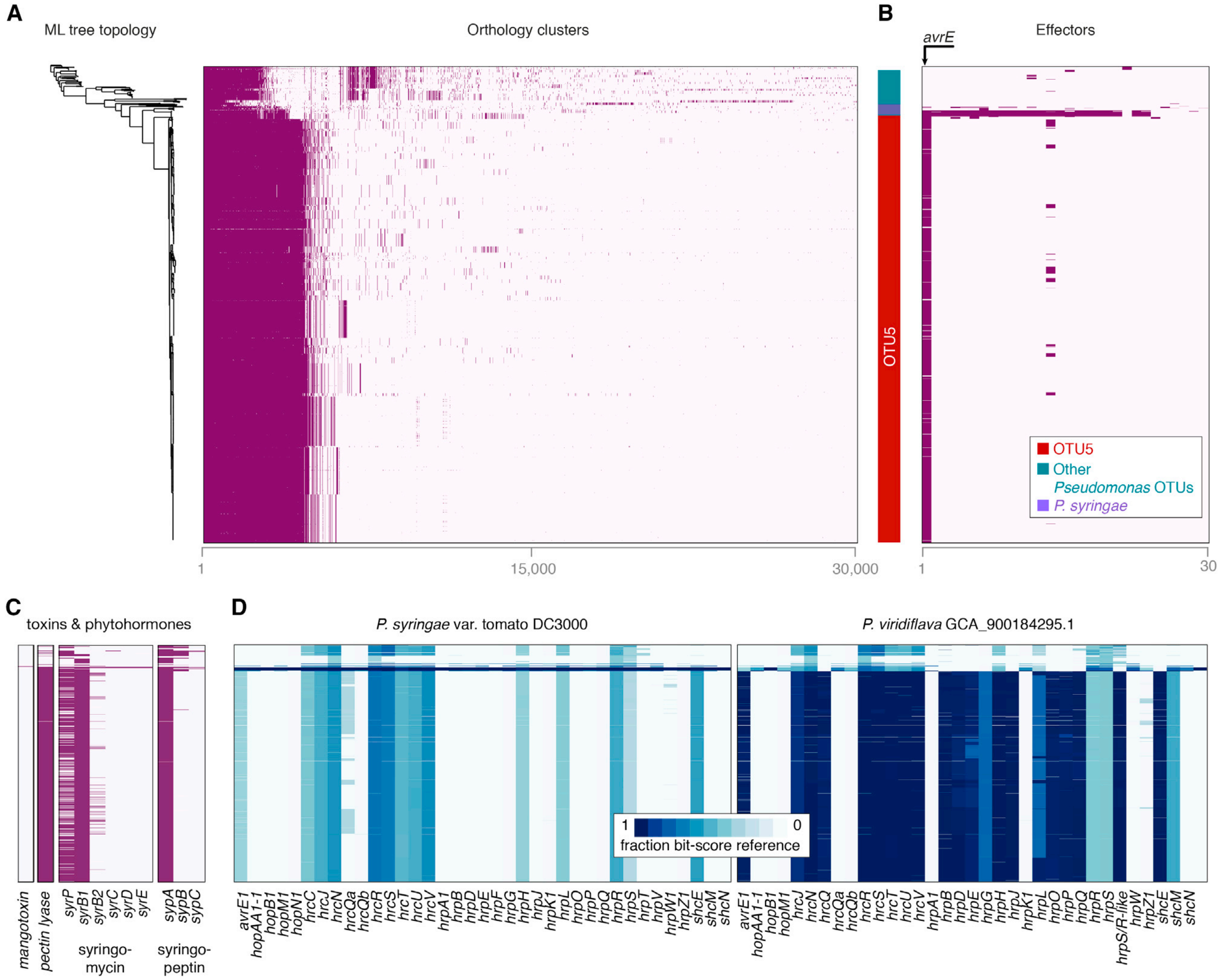

Figure 6. OTU5 Strains Vary in Gene Content but Share an Effector

(A) ML tree topology of 1,524 isolates (for all panels) and presence (dark purple) or absence (light purple) of the 30,000 most common orthologs as inferred with panX (Ding et al., 2018). OTU5 strains share 622 ortholog groups that are found at less than 10% frequency outside OTU5.

(B) Presence/absence of 30 effector homologs. Only *avrE* homologs are present in more than 50 isolates.

(C) Genes for toxins and phytohormones. Only a few genomes contain the full set of genes required for synthesis of syringomycin and syringopeptin.

(D) Similarity of *avrE* and *hrp-hrc* genes to Pto DC3000 and the most similar *P. viridiflava* genome from NCBI. Higher values indicate greater similarity.

Related to Table S2.

We have conducted a large-scale survey of *A. thaliana* leaves to determine local and seasonal diversity of *Pseudomonas*, which includes important *A. thaliana* foliar pathogens. While we found a single OTU to be by far the most abundant, this lineage, OTU5, is nevertheless genetically diverse and consists of dozens, if not hundreds, of strains, diverged by approximately 300,000 years, with similar abilities to colonize the *A. thaliana* host. We were surprised to find a single dominant *Pseudomonas* lineage in the study area, given that wild *A. thaliana* populations can be colonized by a diversity of *Pseudomonas* pathogenic species. We note, though, that while the OTU5 strains share many genetic features, they are not necessarily functionally synonymous—instead, they are differentiated both at the level of gene content and the level of pathogenicity in our laboratory assay. An important question for the future will be in how many other geographic regions OTU5 is the dominant colonizer of *A. thaliana* and how its genetic diversity is structured across the entire host range.

At first glance, the genetic diversity of OTU5 seems to stand in stark contrast to the monomorphic, recent pathogen spreads observed in typical agricultural epidemic systems. There are several non-mutually exclusive explanations for this. Industrial agricultural fields are often planted with one or a few closely related crop genotypes, and environmental variation in these fields is reduced by fertilization prior to planting. The resulting uniformity of the field and host environment is known to influence the microbiota (Figuerola et al., 2015), and to promote the expansions of single pathogens (Zhu et al., 2000). Another

possibility is that the diverse pathogenic expansions we observed also occur in crop populations, but that such expansions may have gone unnoticed because their impact may be small in comparison to the monomorphic crop epidemics. Both theory (Leggett et al., 2013; Regoes et al., 2000) and observations (Ebert, 1998) have detailed scenarios in which specialized pathogens (such as those on crops) will proliferate to higher abundance in their hosts than will generalist pathogens. The approach we have taken in this study, synthesizing microbiome data with single strain and pathogenicity data, provides information on many strains simultaneously. Such data allow for the detection of the population dynamics of strains from a range of abundances and virulences. Future work in crop systems using approaches similar to the ones we have employed here will help to discriminate the dynamics of agricultural versus natural pathosystems.

Most studies of crop pathogen evolution have centered on the loss or gain of a single or a few virulence factors that subvert recognition by the host. Many instances of rapid turnover of virulence factors have been documented (Baltrus et al., 2011; Jackson et al., 2000), even within the span of a few dozen generations. In contrast, in the *P. viridiflava-A. thaliana* system, we observe long-term stability of the *avrE* effector gene. Beyond the molecular mechanism of *avrE*-dependent virulence, a growing number of studies with several pathogens and their plants hosts have demonstrated that *avrE* may be a central determinant for infection success. *avrE* homologs have not only been found in *Pseudomonas*, but have also been identified in other bacterial taxa, where they have been implicated in pathogenicity as well. DspE, an AvrE homolog in the plant pathogen *Erwinia amylovora*, functions similarly to AvrE (Bogdanove et al., 1998), pointing to many pathogens relying on the AvrE mechanism to enhance their fitness. Hosts often have evolved means to detect effector proteins. While several soybean cultivars can recognize the activity of AvrE (Kobayashi et al., 1989), gene-for-gene resistance to the *avrE*-containing Pst DC3000 model pathogen has so far not been found in *A. thaliana* (Velásquez et al., 2017).

It is reasonable to hypothesize that the host has evolved mechanisms that suppress the disease effect of OTU5. By itself, many OTU5 strains can reduce plant growth in gnotobiotic culture by more than 50% or even kill the plant. In natural populations, pathogenic effects appear to be mitigated, since we isolated OTU5 strains from plants that did not appear to be heavily diseased. Indeed, several environmental and genetic factors are known to affect the pathogenic effect of microbes, including the physiological state of the plant (MacQueen and Bergelson, 2016) and the presence of other microbiota (Mendes et al., 2011). Understanding mechanisms of disease mitigation in response to OTU5 will provide insight into how natural plant populations can blunt the effects of a common pathogen without instigating an arms race, and thereby suggest possible innovations to approaching disease protection in agriculture.

STAR★METHODS

Detailed methods are provided in the online version of this paper and include the following:

- KEY RESOURCES TABLE
- CONTACT FOR REAGENT AND RESOURCE SHARING
- EXPERIMENTAL MODEL AND SUBJECT DETAILS
  - Sample Collection
  - Pathogenicity Assays
- METHOD DETAILS
  - 16S v3-v4 Amplicon Sequencing
  - Metagenomics
  - Whole-Genome Sequencing
- QUANTIFICATION AND STATISTICAL ANALYSIS
  - 16S rDNA v3-v4 Amplicon Data Analysis
  - Metagenomic Assessment of Bacterial Load
  - Assembly and Annotation
  - Pan-Genome Analysis and Phylogenetics
- DATA AND SOFTWARE AVAILABILITY
- ADDITIONAL RESOURCES

SUPPLEMENTAL INFORMATION

Supplemental Information includes six figures and two tables and can be found with this article online at https://doi.org/10.1016/j.chom.2018.06.011.

ACKNOWLEDGMENTS

We thank Marek Kucka for methodological help with Tn5 transpososome purification; Julia Vorholt for recommendations on infection methods; Matthew Clark for providing protocols for amended Nextera methodology; Mathew Agler for organizing and participating in several sampling trips; Hernán Burbano for discussions; Joy Bergelson, Jeff Dangl, and Michael Werner for critical reading of the manuscript; and Joffrey Fitz for help with the panX visualization. Funding was provided by HFSP long-term fellowships (LT000348/2016-L, T.L.K.; LT000565/2015-L, D.S.L.), an EMBO long-term fellowship (LRTF 1483-2015, T.L.K.), ERC AdG IMMUNEMESIS (D.W.), and the Max Planck Society (D.W. and E.K.).

AUTHOR CONTRIBUTIONS

T.L.K., E.K., and D.W. devised the study. T.L.K., J.A., C.F., M.G., S.K., D.S.L., and M.N. performed the experiments, and T.L.K., J.A., W.D., D.S.L., M.G., J.R., and R.A.N. analyzed the data. D.H. advised on library preparation methods. R.A.N., E.K., and D.W. advised on data analysis. T.L.K., J.A., and D.W. wrote the manuscript with help from all authors.

DECLARATION OF INTERESTS

The authors declare no competing interests.

Received: January 29, 2018
Revised: May 16, 2018
Accepted: June 21, 2018
Published: July 11, 2018

REFERENCES

1001 Genomes Consortium (2016). 1,135 genomes reveal the global pattern of polymorphism in *Arabidopsis thaliana*. Cell *166*, 481–491.

Agler, M.T., Ruhe, J., Kroll, S., Morhenn, C., Kim, S.-T., Weigel, D., and Kemen, E.M. (2016). Microbial hub taxa link host and abiotic factors to plant microbiome variation. PLoS Biol. *14*, e1002352.

Alfano, J.R., Charkowski, A.O., Deng, W.L., Badel, J.L., Petnicki-Ocwieja, T., van Dijk, K., and Collmer, A. (2000). The *Pseudomonas syringae Hrp* pathogenicity island has a tripartite mosaic structure composed of a cluster of type III secretion genes bounded by exchangeable effector and conserved effector loci that contribute to parasitic fitness and pathogenicity in plants. Proc. Natl. Acad. Sci. USA *97*, 4856–4861.

Almario, J., Jeena, G., Wunder, J., Langen, G., Zuccaro, A., Coupland, G., and Bucher, M. (2017). Root-associated fungal microbiota of nonmycorrhizal *Arabis alpina* and its contribution to plant phosphorus nutrition. Proc. Natl. Acad. Sci. USA *114*, E9403–E9412.

Altschul, S.F., Gish, W., Miller, W., Myers, E.W., and Lipman, D.J. (1990). Basic local alignment search tool. J. Mol. Biol. *215*, 403–410.

Anderson, R.M., and May, R.M. (1982). Coevolution of hosts and parasites. Parasitology *85*, 411–426.

Araki, H., Tian, D., Goss, E.M., Jakob, K., Halldorsdottir, S.S., Kreitman, M., and Bergelson, J. (2006). Presence/absence polymorphism for alternative pathogenicity islands in *Pseudomonas viridiflava*, a pathogen of Arabidopsis. Proc. Natl. Acad. Sci. USA *103*, 5887–5892.

Bakken, J.S., Borody, T., Brandt, L.J., Brill, J.V., Demarco, D.C., Franzos, M.A., Kelly, C., Khoruts, A., Louie, T., Martinelli, L.P., et al.; Fecal Microbiota Transplantation Workgroup (2011). Treating *Clostridium difficile* infection with fecal microbiota transplantation. Clin. Gastroenterol. Hepatol. *9*, 1044–1049.

Balestra, G.M., Mazzaglia, A., Quattrucci, A., Renzi, M., and Rossetti, A. (2009). Current status of bacterial canker spread on kiwifruit in Italy. Australas. Plant Dis. Notes *4*, 34–36.

Baltrus, D.A., Nishimura, M.T., Romanchuk, A., Chang, J.H., Mukhtar, M.S., Cherkis, K., Roach, J., Grant, S.R., Jones, C.D., and Dangl, J.L. (2011). Dynamic evolution of pathogenicity revealed by sequencing and comparative genomics of 19 *Pseudomonas syringae* isolates. PLoS Pathog. *7*, e1002132.

Baltrus, D.A., Nishimura, M.T., Dougherty, K.M., Biswas, S., Mukhtar, M.S., Vicente, J., Holub, E.B., and Dangl, J.L. (2012). The molecular basis of host specialization in bean pathovars of *Pseudomonas syringae*. Mol. Plant Microbe Interact. *25*, 877–888.

Bankevich, A., Nurk, S., Antipov, D., Gurevich, A.A., Dvorkin, M., Kulikov, A.S., Lesin, V.M., Nikolenko, S.I., Pham, S., Prjibelski, A.D., et al. (2012). SPAdes: a new genome assembly algorithm and its applications to single-cell sequencing. J. Comput. Biol. *19*, 455–477.

Barrett, L.G., Kniskern, J.M., Bodenhausen, N., Zhang, W., and Bergelson, J. (2009). Continua of specificity and virulence in plant host-pathogen interactions: causes and consequences. New Phytol. *183*, 513–529.

Barrett, L.G., Bell, T., Dwyer, G., and Bergelson, J. (2011). Cheating, trade-offs and the evolution of aggressiveness in a natural pathogen population. Ecol. Lett. *14*, 1149–1157.

Bartoli, C., Berge, O., Monteil, C.L., Guilbaud, C., Balestra, G.M., Varvaro, L., Jones, C., Dangl, J.L., Baltrus, D.A., Sands, D.C., and Morris, C.E. (2014). The *Pseudomonas viridiflava* phylogroups in the *P. syringae* species complex are characterized by genetic variability and phenotypic plasticity of pathogenicity-related traits. Environ. Microbiol. *16*, 2301–2315.

Baym, M., Kryazhimskiy, S., Lieberman, T.D., Chung, H., Desai, M.M., and Kishony, R. (2015). Inexpensive multiplexed library preparation for megabase-sized genomes. PLoS One *10*, e0128036.

Bogdanove, A.J., Kim, J.F., Wei, Z., Kolchinsky, P., Charkowski, A.O., Conlin, A.K., Collmer, A., and Beer, S.V. (1998). Homology and functional similarity of an *hrp*-linked pathogenicity locus, *dspEF*, of *Erwinia amylovora* and the avirulence locus *avrE* of *Pseudomonas syringae* pathovar tomato. Proc. Natl. Acad. Sci. USA *95*, 1325–1330.

Bomblies, K., Yant, L., Laitinen, R.A., Kim, S.-T., Hollister, J.D., Warthmann, N., Fitz, J., and Weigel, D. (2010). Local-scale patterns of genetic variability, outcrossing, and spatial structure in natural stands of *Arabidopsis thaliana*. PLoS Genet. *6*, e1000890.

Brown, K.A., Khanafer, N., Daneman, N., and Fisman, D.N. (2013). Meta-analysis of antibiotics and the risk of community-associated *Clostridium difficile* infection. Antimicrob. Agents Chemother. *57*, 2326–2332.

Buchfink, B., Xie, C., and Huson, D.H. (2015). Fast and sensitive protein alignment using DIAMOND. Nat. Methods *12*, 59–60.

Buell, C.R., Joardar, V., Lindeberg, M., Selengut, J., Paulsen, I.T., Gwinn, M.L., Dodson, R.J., Deboy, R.T., Durkin, A.S., Kolonay, J.F., et al. (2003). The complete genome sequence of the *Arabidopsis* and tomato pathogen *Pseudomonas syringae* pv. *tomato* DC3000. Proc. Natl. Acad. Sci. USA *100*, 10181–10186.

Butler, M.I., Stockwell, P.A., Black, M.A., Day, R.C., Lamont, I.L., and Poulter, R.T.M. (2013). *Pseudomonas syringae* pv. *actinidiae* from recent outbreaks of kiwifruit bacterial canker belong to different clones that originated in China. PLoS One *8*, e57464.

Cai, R., Lewis, J., Yan, S., Liu, H., Clarke, C.R., Campanile, F., Almeida, N.F., Studholme, D.J., Lindeberg, M., Schneider, D., et al. (2011). The plant pathogen *Pseudomonas syringae* pv. *tomato* is genetically monomorphic and under strong selection to evade tomato immunity. PLoS Pathog. *7*, e1002130.

Caruccio, N. (2011). Preparation of next-generation sequencing libraries using Nextera™ technology: simultaneous DNA fragmentation and adaptor tagging by in vitro transposition. Methods Mol. Biol. *733*, 241–255.

Chen, L.-Q., Hou, B.-H., Lalonde, S., Takanaga, H., Hartung, M.L., Qu, X.-Q., Guo, W.-J., Kim, J.-G., Underwood, W., Chaudhuri, B., et al. (2010). Sugar transporters for intercellular exchange and nutrition of pathogens. Nature *468*, 527–532.

Clarke, C.R., Cai, R., Studholme, D.J., Guttman, D.S., and Vinatzer, B.A. (2010). *Pseudomonas syringae* strains naturally lacking the classical *P. syringae hrp/hrc* locus are common leaf colonizers equipped with an atypical type III secretion system. Mol. Plant Microbe Interact. *23*, 198–210.

DeAngelis, M.M., Wang, D.G., and Hawkins, T.L. (1995). Solid-phase reversible immobilization for the isolation of PCR products. Nucleic Acids Res. *23*, 4742–4743.

Didelot, X., and Wilson, D.J. (2015). ClonalFrameML: efficient inference of recombination in whole bacterial genomes. PLoS Comput. Biol. *11*, e1004041.

Dillion, M.M., Thakur, S., Almeida, R.N.D., and Guttman, D.S. (2017). Recombination of ecologically and evolutionarily significant loci maintains genetic cohesion in the *Pseudomonas syringae* species complex. bioRxiv. https://doi.org/10.1101/227413.

Ding, W., Baumdicker, F., and Neher, R.A. (2018). panX: pan-genome analysis and exploration. Nucleic Acids Res. *46*, e5.

Dray, S., and Dufour, A.-B. (2007). The ade4 package: implementing the duality diagram for ecologists. J. Stat. Softw. *22*, 1–20.

Duchmann, R., Kaiser, I., Hermann, E., Mayet, W., Ewe, K., and Meyer zum Büschenfelde, K.H. (1995). Tolerance exists towards resident intestinal flora but is broken in active inflammatory bowel disease (IBD). Clin. Exp. Immunol. *102*, 448–455.

Ebert, D. (1998). Experimental evolution of parasites. Science *282*, 1432–1435.

Exposito-Alonso, M., Becker, C., Schuenemann, V.J., Reiter, E., Setzer, C., Slovak, R., Brachi, B., Hagmann, J., Grimm, D.G., Chen, J., et al. (2018). The rate and potential relevance of new mutations in a colonizing plant lineage. PLoS Genet. *14*, e1007155.

Falkinham, J.O., 3rd, Hilborn, E.D., Arduino, M.J., Pruden, A., and Edwards, M.A. (2015). Epidemiology and ecology of opportunistic premise plumbing pathogens: *Legionella pneumophila, Mycobacterium avium*, and *Pseudomonas aeruginosa*. Environ. Health Perspect. *123*, 749–758.

Figuerola, E.L.M., Guerrero, L.D., Türkowsky, D., Wall, L.G., and Erijman, L. (2015). Crop monoculture rather than agriculture reduces the spatial turnover of soil bacterial communities at a regional scale. Environ. Microbiol. *17*, 678–688.

Gómez-Gómez, L., Felix, G., and Boller, T. (1999). A single locus determines sensitivity to bacterial flagellin in *Arabidopsis thaliana*. Plant J. *18*, 277–284.

Gomila, M., Peña, A., Mulet, M., Lalucat, J., and García-Valdés, E. (2015). Phylogenomics and systematics in *Pseudomonas*. Front. Microbiol. *6*, 214.

Hedge, J., and Wilson, D.J. (2014). Bacterial phylogenetic reconstruction from whole genomes is robust to recombination but demographic inference is not. MBio *5*, e02158.

Hill, M.O. (1973). Diversity and evenness: a unifying notation and its consequences. Ecology *54*, 427–432.

Hu, J., Wei, Z., Friman, V.-P., Gu, S.-H., Wang, X.-F., Eisenhauer, N., Yang, T.-J., Ma, J., Shen, Q.-R., Xu, Y.-C., and Jousset, A. (2016). Probiotic diversity

enhances rhizosphere microbiome function and plant disease suppression. MBio *7*, e01790–16.

Huson, D.H., Auch, A.F., Qi, J., and Schuster, S.C. (2007). MEGAN analysis of metagenomic data. Genome Res. *17*, 377–386.

Jackson, R.W., Mansfield, J.W., Arnold, D.L., Sesma, A., Paynter, C.D., Murillo, J., Taylor, J.D., and Vivian, A. (2000). Excision from tRNA genes of a large chromosomal region, carrying *avrPphB*, associated with race change in the bean pathogen, *Pseudomonas syringae* pv. *phaseolicola*. Mol. Microbiol. *38*, 186–197.

Jakob, K., Goss, E.M., Araki, H., Van, T., Kreitman, M., and Bergelson, J. (2002). *Pseudomonas viridiflava* and *P. syringae*—natural pathogens of *Arabidopsis thaliana*. Mol. Plant Microbe Interact. *15*, 1195–1203.

Karasov, T.L., Kniskern, J.M., Gao, L., DeYoung, B.J., Ding, J., Dubiella, U., Lastra, R.O., Nallu, S., Roux, F., Innes, R.W., et al. (2014a). The long-term maintenance of a resistance polymorphism through diffuse interactions. Nature *512*, 436–440.

Karasov, T.L., Horton, M.W., and Bergelson, J. (2014b). Genomic variability as a driver of plant-pathogen coevolution? Curr. Opin. Plant Biol. *18*, 24–30.

Karasov, T.L., Barrett, L., Hershberg, R., and Bergelson, J. (2017). Similar levels of gene content variation observed for *Pseudomonas syringae* populations extracted from single and multiple host species. PLoS One *12*, e0184195.

Kimura, M. (1968). Evolutionary rate at the molecular level. Nature *217*, 624–626.

Kniskern, J.M., Barrett, L.G., and Bergelson, J. (2011). Maladaptation in wild populations of the generalist plant pathogen *Pseudomonas syringae*. Evolution *65*, 818–830.

Kobayashi, D.Y., Tamaki, S.J., and Keen, N.T. (1989). Cloned avirulence genes from the tomato pathogen *Pseudomonas syringae* pv. *tomato* confer cultivar specificity on soybean. Proc. Natl. Acad. Sci. USA *86*, 157–161.

Kolmer, J.A. (2005). Tracking wheat rust on a continental scale. Curr. Opin. Plant Biol. *8*, 441–449.

Kryazhimskiy, S., and Plotkin, J.B. (2008). The population genetics of dN/dS. PLoS Genet. *4*, e1000304.

Laine, A.-L., Burdon, J.J., Dodds, P.N., and Thrall, P.H. (2011). Spatial variation in disease resistance: from molecules to metapopulations. J. Ecol. *99*, 96–112.

Leggett, H.C., Buckling, A., Long, G.H., and Boots, M. (2013). Generalism and the evolution of parasite virulence. Trends Ecol. Evol. *28*, 592–596.

Li, H. (2013). Aligning sequence reads, clone sequences and assembly contigs with BWA-MEM. arXiv, arXiv:303.3997, https://arxiv.org/abs/1303.3997.

Li, H., Handsaker, B., Wysoker, A., Fennell, T., Ruan, J., Homer, N., Marth, G., Abecasis, G., and Durbin, R.; 1000 Genome Project Data Processing Subgroup (2009). The Sequence Alignment/Map format and SAMtools. Bioinformatics *25*, 2078–2079.

MacQueen, A., and Bergelson, J. (2016). Modulation of R-gene expression across environments. J. Exp. Bot. *67*, 2093–2105.

McCann, H.C., Li, L., Liu, Y., Li, D., Pan, H., Zhong, C., Rikkerink, E.H.A., Templeton, M.D., Straub, C., Colombi, E., et al. (2017). Origin and evolution of the kiwifruit canker pandemic. Genome Biol. Evol. *9*, 932–944.

Mendes, R., Kruijt, M., de Bruijn, I., Dekkers, E., van der Voort, M., Schneider, J.H.M., Piceno, Y.M., DeSantis, T.Z., Andersen, G.L., Bakker, P.A.H.M., and Raaijmakers, J.M. (2011). Deciphering the rhizosphere microbiome for disease-suppressive bacteria. Science *332*, 1097–1100.

Moeller, A.H., Caro-Quintero, A., Mjungu, D., Georgiev, A.V., Lonsdorf, E.V., Muller, M.N., Pusey, A.E., Peeters, M., Hahn, B.H., and Ochman, H. (2016). Cospeciation of gut microbiota with hominids. Science *353*, 380–382.

Morris, C.E., Sands, D.C., Vanneste, J.L., Montarry, J., Oakley, B., Guilbaud, C., and Glaux, C. (2010). Inferring the evolutionary history of the plant pathogen *Pseudomonas syringae* from its biogeography in headwaters of rivers in North America, Europe, and New Zealand. MBio *1*, e00107–e00110.

Oksanen, J., Blanchet, F., Kindt, R., Legendre, P., O'Hara, B. (2016) Vegan: community ecology package. R Package 2.3-3.

Ondov, B.D., Treangen, T.J., Melsted, P., Mallonee, A.B., Bergman, N.H., Koren, S., and Phillippy, A.M. (2016). Mash: fast genome and metagenome distance estimation using MinHash. Genome Biol. *17*, 132.

Park, D.J., Dudas, G., Wohl, S., Goba, A., Whitmer, S.L.M., Andersen, K.G., Sealfon, R.S., Ladner, J.T., Kugelman, J.R., Matranga, C.B., et al. (2015). Ebola virus epidemiology, transmission, and evolution during seven months in Sierra Leone. Cell *161*, 1516–1526.

Pirnay, J.-P., Bilocq, F., Pot, B., Cornelis, P., Zizi, M., Van Eldere, J., Deschaght, P., Vaneechoutte, M., Jennes, S., Pitt, T., and De Vos, D. (2009). *Pseudomonas aeruginosa* population structure revisited. PLoS One *4*, e7740.

R Development Core Team (2010). R: A Language and Environment for Statistical Computing (R Foundation for Statistical Computing).

Regoes, R.R., Nowak, M.A., and Bonhoeffer, S. (2000). Evolution of virulence in a heterogeneous host population. Evolution *54*, 64–71.

Rocha, E.P.C., Smith, J.M., Hurst, L.D., Holden, M.T.G., Cooper, J.E., Smith, N.H., and Feil, E.J. (2006). Comparisons of dN/dS are time dependent for closely related bacterial genomes. J. Theor. Biol. *239*, 226–235.

Schloss, P.D., Westcott, S.L., Ryabin, T., Hall, J.R., Hartmann, M., Hollister, E.B., Lesniewski, R.A., Oakley, B.B., Parks, D.H., Robinson, C.J., et al. (2009). Introducing mothur: open-source, platform-independent, community-supported software for describing and comparing microbial communities. Appl. Environ. Microbiol. *75*, 7537–7541.

Schmidt, T.M., DeLong, E.F., and Pace, N.R. (1991). Analysis of a marine picoplankton community by 16S rRNA gene cloning and sequencing. J. Bacteriol. *173*, 4371–4378.

Schneider, D.S., and Ayres, J.S. (2008). Two ways to survive infection: what resistance and tolerance can teach us about treating infectious diseases. Nat. Rev. Immunol. *8*, 889–895.

Seemann, T. (2014). Prokka: rapid prokaryotic genome annotation. Bioinformatics *30*, 2068–2069.

Simão, F.A., Waterhouse, R.M., Ioannidis, P., Kriventseva, E.V., and Zdobnov, E.M. (2015). BUSCO: assessing genome assembly and annotation completeness with single-copy orthologs. Bioinformatics *31*, 3210–3212.

Stamatakis, A., Ludwig, T., and Meier, H. (2005). RAxML-III: a fast program for maximum likelihood-based inference of large phylogenetic trees. Bioinformatics *21*, 456–463.

Stanke, M., and Waack, S. (2003). Gene prediction with a hidden Markov model and a new intron submodel. Bioinformatics *19* (*Suppl 2*), ii215–ii225.

Thrall, P.H., Burdon, J.J., and Young, A. (2001). Variation in resistance and virulence among demes of a plant host–pathogen metapopulation. J. Ecol. *89*, 736.

Vandeputte, D., Kathagen, G., D'hoe, K., Vieira-Silva, S., Valles-Colomer, M., Sabino, J., Wang, J., Tito, R.Y., De Commer, L., Darzi, Y., et al. (2017). Quantitative microbiome profiling links gut community variation to microbial load. Nature *551*, 507–511.

Vaughn, D.W., Green, S., Kalayanarooj, S., Innis, B.L., Nimmannitya, S., Suntayakorn, S., Endy, T.P., Raengsakulrach, B., Rothman, A.L., Ennis, F.A., and Nisalak, A. (2000). Dengue viremia titer, antibody response pattern, and virus serotype correlate with disease severity. J. Infect. Dis. *181*, 2–9.

Velásquez, A.C., Oney, M., Huot, B., Xu, S., and He, S.Y. (2017). Diverse mechanisms of resistance to *Pseudomonas syringae* in a thousand natural accessions of *Arabidopsis thaliana*. New Phytol. *214*, 1673–1687.

Walker, B.J., Abeel, T., Shea, T., Priest, M., Abouelliel, A., Sakthikumar, S., Cuomo, C.A., Zeng, Q., Wortman, J., Young, S.K., and Earl, A.M. (2014). Pilon: an integrated tool for comprehensive microbial variant detection and genome assembly improvement. PLoS One *9*, e112963.

Weigel, D. (2012). Natural variation in *Arabidopsis*: from molecular genetics to ecological genomics. Plant Physiol. *158*, 2–22.

Wiehlmann, L., Wagner, G., Cramer, N., Siebert, B., Gudowius, P., Morales, G., Köhler, T., van Delden, C., Weinel, C., Slickers, P., and Tümmler, B. (2007). Population structure of *Pseudomonas aeruginosa*. Proc. Natl. Acad. Sci. USA *104*, 8101–8106.

Woolhouse, M.E., Taylor, L.H., and Haydon, D.T. (2001). Population biology of multihost pathogens. Science *292*, 1109–1112.

Xin, X.-F., Nomura, K., Aung, K., Velásquez, A.C., Yao, J., Boutrot, F., Chang, J.H., Zipfel, C., and He, S.Y. (2016). Bacteria establish an aqueous living space in plants crucial for virulence. Nature *539*, 524–529.

Yoshida, K., Schuenemann, V.J., Cano, L.M., Pais, M., Mishra, B., Sharma, R., Lanz, C., Martin, F.N., Kamoun, S., Krause, J., et al. (2013). The rise and fall of the *Phytophthora infestans* lineage that triggered the Irish potato famine. Elife *2*, e00731.

Yunis, H., Bashan, Y., Okon, Y., and Henis, Y. (1980). Weather dependence, yield losses, and control of bacterial speck of tomato caused by *Pseudomonas tomato*. Plant Dis. *64*, 937–939.

Zhu, Y., Chen, H., Fan, J., Wang, Y., Li, Y., Chen, J., Fan, J., Yang, S., Hu, L., Leung, H., et al. (2000). Genetic diversity and disease control in rice. Nature *406*, 718–722.

STAR★METHODS

KEY RESOURCES TABLE

REAGENT or RESOURCE	SOURCE	IDENTIFIER
Deposited Data		
1524 Pseudomonas genomes	This study	ENA: PRJEB24450
192 A. thaliana metagenomes	This study	ENA: PRJEB24450
192 16S v4 sequences	This study	ENA: PRJEB24450
Sequences of v3-v4 region	This study	SRA: PRJNA430505
Pan-genome data and visualization	This study	http://panx.weigelworld.org/
Experimental Models: Organisms/Strains		
1524 *Pseudomonas* strains	This study	N/A
Pst DC3000	Thorsten Nürnberger	DC3000
36 *Pseudomonas* tested for growth effect	This study	N/A
A. thaliana genotype Ey15-2	*Arabidopsis* Stock Center TAIR	CS76399
Software and Algorithms		
Mash	Ondov et al., 2016	https://github.com/marbl/Mash
Mothur	Schloss et al., 2009	https://github.com/mothur/mothur
RAxML	Stamatakis et al., 2005	https://github.com/stamatak/standard-RAxML
Vegan	Oksanen et al, 2016	https://github.com/vegandevs/vegan

CONTACT FOR REAGENT AND RESOURCE SHARING

Further information and requests for resources and reagents should be directed to and will be fulfilled by the Lead Contact, Detlef Weigel (weigel@tue.mpg.de).

EXPERIMENTAL MODEL AND SUBJECT DETAILS

Sample Collection

For the 16S rDNA survey, *A. thaliana* samples were collected from five to six populations (sites) around Tübingen (Figure S1A), in the fall and spring of 2014, 2015 and 2016; the number of sampled plants is indicated in Figure 1A. For endophytic and epiphytic sample fractionation, whole rosettes were processed as described in Agler et al. (2016). Briefly, rosettes were washed once in water for 30 s, then in 3–5 mL of epiphyte wash solution (0.1% Triton X-100 in 1x TE buffer) for 1 min, before filtering the solution through a 0.2 μm nitrocellulose membrane filter (Whatman, Piscataway, NJ, USA) to collect the epiphytic fraction. For the endophytic fraction, the initial rosette was surface sterilized by washing with 80% ethanol for 15 s followed by 2% bleach (sodium hypochlorite) for 30 s, before rinsing three times with sterile autoclaved water. Samples were stored in screw cap tubes and directly frozen in dry ice. DNA extraction was conducted following Agler et al. (2016), including a manual sample grinding step followed by a lysis step with SDS, Lysozyme and proteinase K, a DNA extraction step based on phenol-chloroform and a final DNA precipitation step with 100% ethanol.

Additional samples were collected from four of the six sites sampled for 16S rDNA, from Eyach (EY), on December 11, 2015, and March 23, 2016, and from Kirchentellinsfurt (JUG) on December 15, 2016, and March 31, 2016, on March 31, 2016 for PFN and April 6, 2016 for K6911. Colonies were isolated only for samples from EY and JUG. Whole rosettes were removed with sterile scissors and tweezers, and washed with deionized water. Two leaves were removed and independently processed, and the remaining rosette was flash-frozen on dry ice. The flash-frozen material was processed for metagenomic sequencing and 16S rDNA sequencing of the v4 region. The removed leaves were placed on ice, washed in 70%–80% EtOH for 3-5 s to remove lightly-associated epiphytes. Sterilized plants were ground in 10 mM $MgSO_4$ and plated on King's Broth (KB) plates containing 100 μg/mL nitrofurantoin (Sigma). Plates were incubated at 25°C for two days, then placed at 4°C. Colonies were picked randomly from plates between 3-10 days after plating, grown in KB with nitrofurantoin overnight, then stored at −80°C in 15%–30% glycerol.

Pathogenicity Assays

The plant genotype Eyach 15-2 (CS76399), collected from Eyach, Germany, was previously determined to represent a plant genetic background common to the geographical region (Bomblies et al., 2010). Seeds were sterilized by overnight incubation at −80°C, followed by 4 hours of bleach treatment at room temperature (seeds in open 2 mL tube in a desiccator containing a beaker with

40 mL Chlorox and 1 mL HCl (32%)). The seeds were stratified for three days at 4°C in the dark on ½ MS media. Plants were grown in 3-4 mL ½ MS medium in six-well plates in long-day (16 hours) at 16°C. 12-14 days after stratification, plants were infected with single bacterial strains.

Bacteria were grown overnight in Luria broth and the relevant antibiotic (either 10 μg/mL of Kanamycin or Nitrofurantoin), diluted 1:10 in the morning and grown for 2 additional hours until they entered log phase. The bacteria were pelleted at 3500 g, resuspended in 10 mM $MgSO_4$ to a concentration of OD_{600} = 0.01. 200 μl of bacteria were drip-inoculated with a pipette onto the whole rosette. Plates were sealed with Parafilm and returned to the growth chamber. Seven days after infection, whole rosettes were cut from the plant and fresh mass was assessed.

For growth assays of dead bacteria, we performed growth and dilution of bacteria as above, then boiled the final preparation at 95°C for 38 minutes. Plants were treated with the dead bacteria in the same manner as described above.

METHOD DETAILS

16S v3-v4 Amplicon Sequencing

The 16S v3-v4 region was amplified as described (Agler et al., 2016). Briefly, PCR reactions were carried out using a two-step protocol using blocking primers to reduce plant plastid 16S rDNA amplification. The first PCR was conducted with primers B341F / B806R in 20 μL reactions containing 0.2 μL Q5 high-fidelity DNA polymerase (New England Biolabs, Ipswich, MA, USA), 1x Q5 GC Buffer, 1x Q5 5x reaction buffer, 0.08 μM each of forward and reverse primer, 0.25 μM blocking primer and 225 μM dNTP. Template DNA was diluted 1:1 in nuclease free water and 1 μL was added to the PCR. Triplicates were run in parallel on three independent thermocyclers (Bio-Rad Laboratories, Hercules, CA, USA); cycling conditions were 95°C for 40 s, 10 cycles of 95°C for 35 s, 55°C for 45 s, 72°C for 15 s, and a final elongation at 72°C for 3 min. The three reactions were combined and 10 μL were used for enzymatic cleanup with Antarctic phosphatase and Exonuclease I (New England Biolabs; 0.5 μL of each enzyme with 1.22 μL Antarctic phosphatase buffer at 37°C for 30 minutes followed by 80°C for 15 min). One microliter of cleaned PCR product was subsequently used in the second PCR with tagged primers including the Illumina adapters, in 50 μL containing 0.5 μL Q5 high-fidelity DNA polymerase (New England Biolabs), 1x Q5 GC Buffer, 1x Q5 5x reaction buffer, 0.16 μM each of forward and reverse primer and 200 μM dNTP. Cycling conditions were the same as for the first PCR except amplification was limited to 25 cycles. The final PCR products were cleaned using 1.8x volume Ampure XP purification beads (Beckman-Coulter, Brea, CA, USA) and eluted in 40 μL according to manufacturer instructions. Amplicons were quantified in duplicates with the PicoGreen system (Life Technologies, Carlsbad, CA, USA) and samples were combined in equimolar amounts into one library. The final libraries were cleaned with 0.8x volume Ampure XP purification beads and eluted into 40 μL. Libraries were prepared with the MiSeq Reagent Kit v3 for 2x300 bp paired-end reads (Illumina, San Diego, CA, USA) with 3% PhiX control. All the samples were analyzed in 9 runs on the same Illumina MiSeq instrument. Samples failing to produce enough reads on one run were re-sequenced and data from both runs were merged.

Metagenomics

Total DNA was extracted from flash-frozen rosettes by pre-grinding the frozen plant material to a powder using a mortar and pestle lined with sterile (autoclaved) aluminum foil and liquid nitrogen as needed to keep the sample frozen. Between 100 mg and 200 mg of plant material were then transferred with a sterile spatula to a 2 mL screw cap tube (Sarstedt) containing 0.5 mL of 1 mM garnet rocks (BioSpec). To this, 800 μL of room temperature extraction buffer was added, containing 10 mM Tris pH 8.0, 10 mM EDTA, 100 mM NaCl, and 1.5% SDS. Lysis was performed in a FastPrep homogenizer at speed 6.0 for 1 minute. These tubes were spun at 20,000 x g for 5 minutes, and the supernatant was mixed with $^1/_3$ volume of 5M KOAc in new tubes to precipitate the SDS. This precipitate was in turn spun at 20,000 x g for 5 minutes and DNA was purified from the resulting supernatant using Solid Phase Reversible Immobilisation (SPRI) beads (DeAngelis et al., 1995) at a bead to sample ratio of 1:2. DNA was quantified by PicoGreen, and libraries were constructed using a Nextera protocol modified to include smaller volumes (similar to Baym et al., 2015). Library molecules were size selected on a Blue Pippin instrument (Sage Science, Beverly, MA, USA). Multiplexed libraries were sequenced with 2x150 bp paired-end reads on an HiSeq3000 instrument (Illumina).

Whole-Genome Sequencing

Bacterial DNA, both genomic and plasmid, was extracted using the Puregene DNA extraction kit (Invitrogen). Single bacterial colonies were grown overnight in Luria broth+100 μg/mL Nitrofurantoin in 96-well plates. Plates were spun down for 10 minutes at 8000 g, then the standard Puregene extraction protocol was followed. The capacity of the protocol to extract plasmid DNA was verified by extracting the DNA from a strain whose plasmids were previously identified (Pst DC3000) (Buell et al., 2003). Primers specific to these plasmids successfully amplified the puregene-extracted sample.

Genomic and plasmid DNA libraries for single bacteria and for whole plant metagenomes were constructed using a modified version of the Nextera protocol (Caruccio, 2011), modified to include smaller volumes. Briefly, 0.25-2ng of extracted DNA was sheared with the Nextera Tn5 transposase. Sheared DNA was amplified with custom primers for 14 cycles. Libraries were pooled and size-selected for the 300-700bp range on a Blue Pippin. Resulting libraries were then sequenced on the Illumina HiSeq3000. Coverage and assembly statistics are detailed in Figure S2.

QUANTIFICATION AND STATISTICAL ANALYSIS

16S rDNA v3-v4 Amplicon Data Analysis

Amplicon data analysis was conducted in Mothur (Schloss et al., 2009). Paired-end reads were assembled (*make.contigs*) and reads with fewer than 5 bp overlap (full match) between the forward and reverse reads were discarded (*screen.seqs*). Reads were demultiplexed, filtered to a maximum of two mismatches with the tag sequence and a minimum of 100 bp in length. Chimeras were identified using Uchime in Mothur with more abundant sequences as reference (*chimera.uchime*, abskew = 1.9). Sequences were clustered into OTUs at the 99% similarity threshold using VSEARCH in Mothur with the distance based clustering method (dgc) (*cluster*). Individual sequences were taxonomically classified using the rdp classifier method (*classify.seqs*, consensus confidence threshold set to 80) and the greengenes 16S rDNA database (13_8 release) including the phiX genome (NC_001422.1) to improve the detection of remaining phiX reads. Each OTU was taxonomically classified (*classify.otu,* consensus confidence threshold set to 66), non-bacterial OTUs and OTUs with unknown taxonomy at the kingdom level were removed, as were low abundance OTUs (< 50 reads, *split.abund*). The confidence of OTU classification to the genus *Pseudomonas* was at least 97%. The most abundant sequence within each *Pseudomonas* OTU was selected as the OTU representative for phylogenetic analyses.

All statistical analyses were conducted in R 3.2.3 (R Development Core Team, 2010). In order to avoid zero values, relative abundance data was transformed using a log (x+a) formula where a is the minimum value of the variable divided by two. Normality after transformation was assessed using Shapiro Wilk's normality test. Factors influencing *Pseudomonas* relative abundance were studied using multi-factorial ANOVA. When necessary, sites PFN and K6911 were excluded from the analysis, as they had missing data points (Figure 1A). Mean differences were further verified with Wilcoxon's non-parametric test. Differences between *Pseudomonas* populations were assessed by calculating Bray-Curtis dissimilarities between samples using the "vegdist" function of the vegan package (Oksanen et al., 2016). These distances were used for principal coordinates analysis using the "dudi.pco" function of the ADE4 package (Dray and Dufour, 2007), and for PERMANOVA to study the effect of different factors on the structure of *Pseudomonas* populations using the "Adonis" function of the vegan package.

The 16S rDNA analysis of 176 plants for which metagenomic shotgun data were also generated (see below) involved the amplification of the v4 region using the published primers 515F-806R (Schmidt et al., 1991) on an Illumina Miseq instrument with 2x250 bp paired end reads, which were subsequently merged. Sequences were then processed according to the same pipeline used for the v3-v4 region analysis. One OTU, Otu000002 aligned with 100% identity over its entire length to OTU5, the most abundant OTU identified in the cross-population survey of the v3-v4 region described above.

Metagenomic Assessment of Bacterial Load

A significant challenge in the analysis of plant metagenomic sequences, is the proper removal of the host DNA. In order to remove host derived sequences, reads were mapped against the *A. thaliana* TAIR10 reference genome with bwa mem (Li, 2013) using standard parameters. Subsequently, all read pairs flagged as unmapped were isolated from the main sequencing library with samtools (Li et al., 2009) as this represents the putatively "metagenomic" fraction.

Afterward, this metagenomic fraction was mapped against the NCBI nr database (NCBI Resource Coordinators. Database resources of the National Center for Biotechnology Information 2016) with the blastx implementation of DIAMOND (Buchfink et al., 2015) using standard parameters.

Based on the reference sequences for which our metagenomic reads had significant alignments, taxonomic binning of sequencing data was performed with MEGAN via the naive LCA algorithm (Huson et al., 2007). Normalization of binned reads was performed with custom scripts and based on the number of reads binned into any given genus including reads assigned to species in that genus, taxa abundance was estimated.

Assembly and Annotation

Genomes were assembled using Spades (Bankevich et al., 2012) (standard parameters) and assembly errors corrected using pilon (Walker et al., 2014) (standard parameters). Gene annotations were achieved using Prokka (Seemann, 2014) (standard parameters). Those genomes with N50 < 25kbp or less than 3000 annotated genes were deemed to be of insufficient quality and were excluded from further analyses except for 19 genomes sequenced in the second season. Distributions of gene number and assembly quality are displayed in Figure S3. The number of missing genes per genome was assessed using Busco (Simão et al., 2015).

Because Prokka does not successfully identify several effectors, in addition to other genes involved in interactions with the host, we augmented the Prokka annotation with several additional annotation sets. We predicted genes on the raw genome FASTA sequences using AUGUSTUS-3.3 (Stanke and Waack, 2003) and –genemodel = partial –gff3 = on –species = E_coli_K12 settings. The protein sequence of each predicted gene was extracted using a custom script.

We annotated effectors using BLASTP-2.2.31+ (Altschul et al., 1990) specifying the AUGUSTUS predicted proteomes as query input and the Hop database (http://www.Pseudomonas-syringae.org/T3SS-Hops.xls) as reference database. We filtered the BLASTP results with a 40% identity query to reference sequence threshold, a 60% alignment length threshold of query to reference sequence and a 60% length ratio threshold of query and reference sequence (empirically determined). Hits of interest were manually extracted and controlled using online BLASTP and NCBI conserved domain search.

Toxins and phytohormones were annotated using the same BLASTP settings as described for effectors. We used custom NCBI protein databases including a set of genes involved in the toxin synthesis pathway. A strain was scored as toxin pathway encoding if

all selected components of a pathway were present. Hrp-hrc clusters were also annotated using the formerly described BLAST and filtering settings and *P. syringae pv tomato DC3000* and *P. viridiflava PNA 3.3* as reference sequences.

Pan-Genome Analysis and Phylogenetics

The panX pan-genome pipeline was used to assign orthology clusters (Ding et al., 2018) and build alignments of these clusters that were then used for phylogenetic analysis in RAxML (Stamatakis et al., 2005). The parameters used were the following: divide-and-conquer algorithm (-dmdc) was used on the diamond clustering, a subset size of 50 was used in the dmdc (-dcs 50), a core genome cutoff of 70% (-cg 0.7).

Core-genome phylogenies of the strains were constructed using RAxML (Stamatakis et al., 2005) using the gamma model of rate heterogeneity and the generalized time reversible model of substitution. The phylogenies were built from all sites present in the concatenated core genomes of strains identified by panX. Sites were excluded if there were gaps in 5% or more of the strains. Nine hundred and thirty nine genes were considered as core. We performed 100 bootstrap replicates in RAxML to establish the confidence in the full tree.

Within the 1355 isolates belonging to OTU5, 82 distinct strains were represented. A representative of each of 82 strains of was picked at random in addition to 25 repeated isolates, then recombination importation events were identified among these 107 isolates using ClonalFrameML (Didelot and Wilson, 2015). ClonalFrameML estimated a high recombination rate within OTU5, estimating that a substitution in the tree was six times more likely to result from a recombination event than a mutation event. Specifically, ClonalFrameML estimated the following parameters: the $1/\delta$ parameter (inverse importation event tract length in bp) was estimated as $7.79\text{x}10^{-3}$/bp (var = 2.18014^{-9}) and the Posterior Mean ratio between the probability of recombination (R) and the nucleotide diversity, θ, was $R/\theta = 1.19$ (var = $5.07\text{x}10^{-5}$). The estimated sequence divergence between imported tracts and the acceptor genome $\nu = 0.04$ (var = $1.13\text{x}10^{-8}$). The relative effect of recombination over mutation $r/m = (R/\ \theta)\ \text{x}\ \nu\ \text{x}\ \delta = 6.18$. Predicted recombination tracts were removed from the alignments, and the remaining putatively non-recombined strict core genes (present in all 107 genomes) were used for subsequent dating of coalescence.

To estimate the age of OTU5 we considered only those ortholog groups that were conserved across all 107 OTU5 isolates. These orthologs were concatenated and ClonalFrameML (Didelot and Wilson, 2015) was used to identify recombination tracts that could inflate the branch length of members of the OTU as described above. TMRCA of the OTU was estimated by calculating the mid-point-root to tip sequence divergence for a representative of all 107 strains within OTU5, then dividing the median value of this distance by the neutral substitution rate (Kimura, 1968) (we used here the point estimate of $8.7\text{x}10^{-8}$ with our estimate of sd = $6.0\text{x}10^{-8}$; McCann et al., 2017). While we consider all sites (degenerate and non-degenerate) in the putatively non-recombined core, in addition to the fact that substitution rate is likely inaccurate for the longer timescale analyzed in the present study, both of these inaccuracies would likely lead to the underestimation of the age.

Comparisons of OTU5 strains to reference Pseudomonas genomes were performed using mash (Ondov et al., 2016). A representative genome from each *Pseudomonas* species for which a genome was available in GenBank as of April 2018 was included in a general reference database. Minhash dimensionality reduction was used to calculate the pairwise mutation distance between each of 25 randomly selected OTU5 genomes and those of the reference database.

DATA AND SOFTWARE AVAILABILITY

The raw v3-v4 16S sequencing data was deposited at the National Center for Biotechnology Information (NCBI) Short Read Archive under BioProject SRA: PRJNA430505. Metagenomic short read sequences, v4 16S sequences data and assembled genomes were deposited in the European Nucleotide Archive (ENA) under the Primary Accession ENA: PRJEB24450. Additional code and phylogenies related to the processing of the full genomes is available at: https://github.com/tkarasov/Pseudomonas_1524.

ADDITIONAL RESOURCES

A visualization of the pan-genome, gene-specific and whole genome phylogenies and basic population statistics for the pan-genome of the 1,524 sequenced strains can be found at http://panx.weigelworld.org/.

Cell Host & Microbe, Volume *24*

Supplemental Information

Arabidopsis thaliana and *Pseudomonas* Pathogens Exhibit Stable Associations over Evolutionary Timescales

Talia L. Karasov, Juliana Almario, Claudia Friedemann, Wei Ding, Michael Giolai, Darren Heavens, Sonja Kersten, Derek S. Lundberg, Manuela Neumann, Julian Regalado, Richard A. Neher, Eric Kemen, and Detlef Weigel

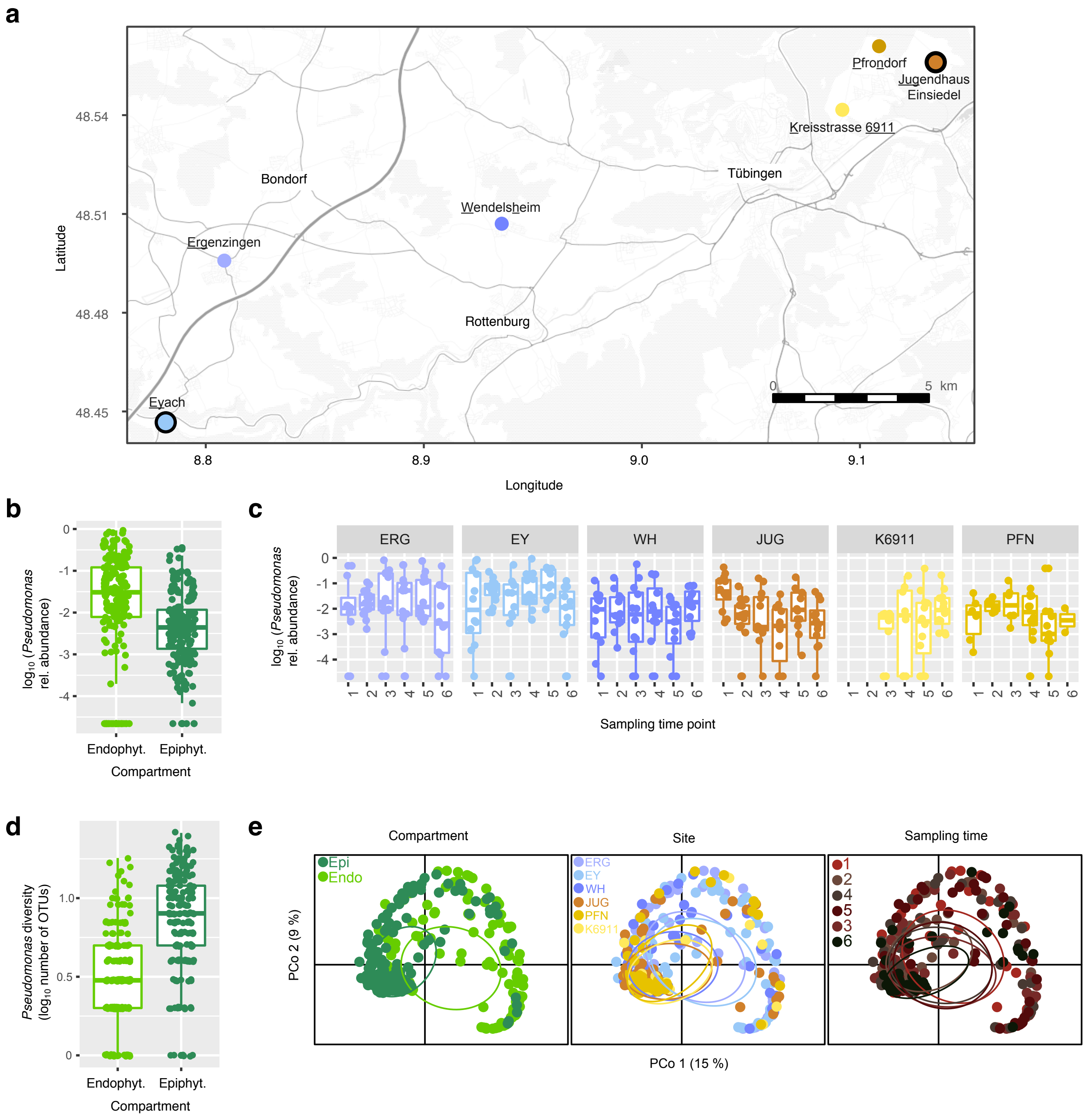

Fig. S1. Changes in *Pseudomonas* populations colonizing *A. thaliana* leaves. Related to Fig. 1. (a) Location of the sampling sites around Tübingen (Germany). The two sites from which isolates were cultured indicated by black outlines. (b) *Pseudomonas* abundance in the endophytic (Endo.) and epiphytic (Epi.) compartments. (c) *Pseudomonas* abundance across the different sites and sampling time points (see Fig. 1a). (d) *Pseudomonas* diversity in the endophytic (Endo.) and epiphytic (Epi.) compartments. (e) Principal coordinates analysis (PCoA) based on Bray-Curtis distances, depicting the differences between *Pseudomonas* populations across the different compartments, sites and sampling time points. *Pseudomonas* relative abundance (RA) was calculated as the ratio of *Pseudomonas* reads to the total number of bacterial reads.

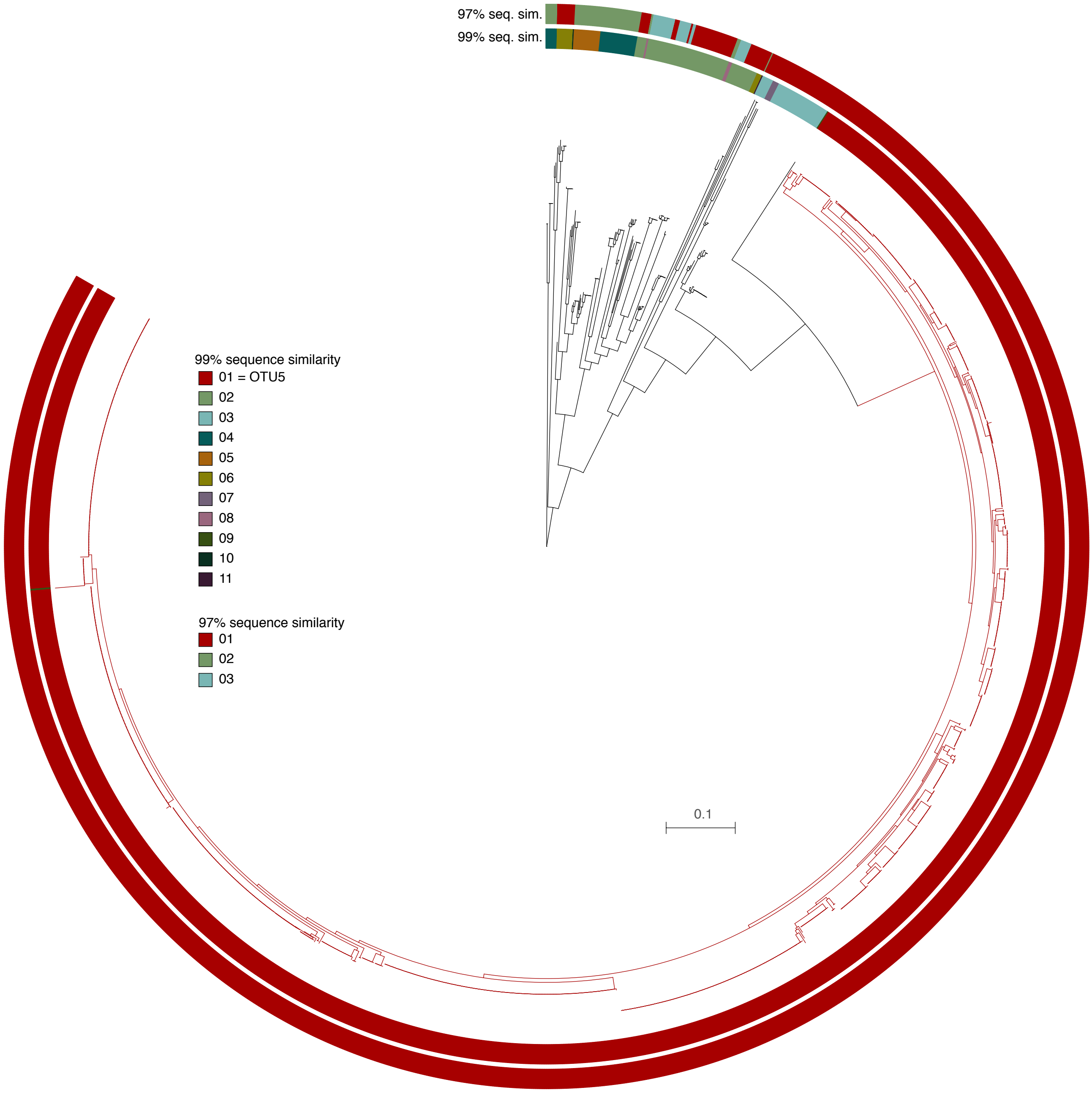

Fig. S2. Pseudomonas OTUs and core-genome phylogeny. Related to Fig. 1, 2 and 3. 16S rDNA sequences were extracted from 1,524 Pseudomonas isolates with core-genome sequences. Clustering based on 99% or 97% sequence identity of the v3-v4 region of the 16S rDNA is compared to an ML core genome phylogenetic tree. Colors indicate group, ranked by abundance. The most abundant group corresponds to OTU5 (Bordeaux color), with clustering at 99% sequence identity being more consistent with the core genome tree than 97% clustering.

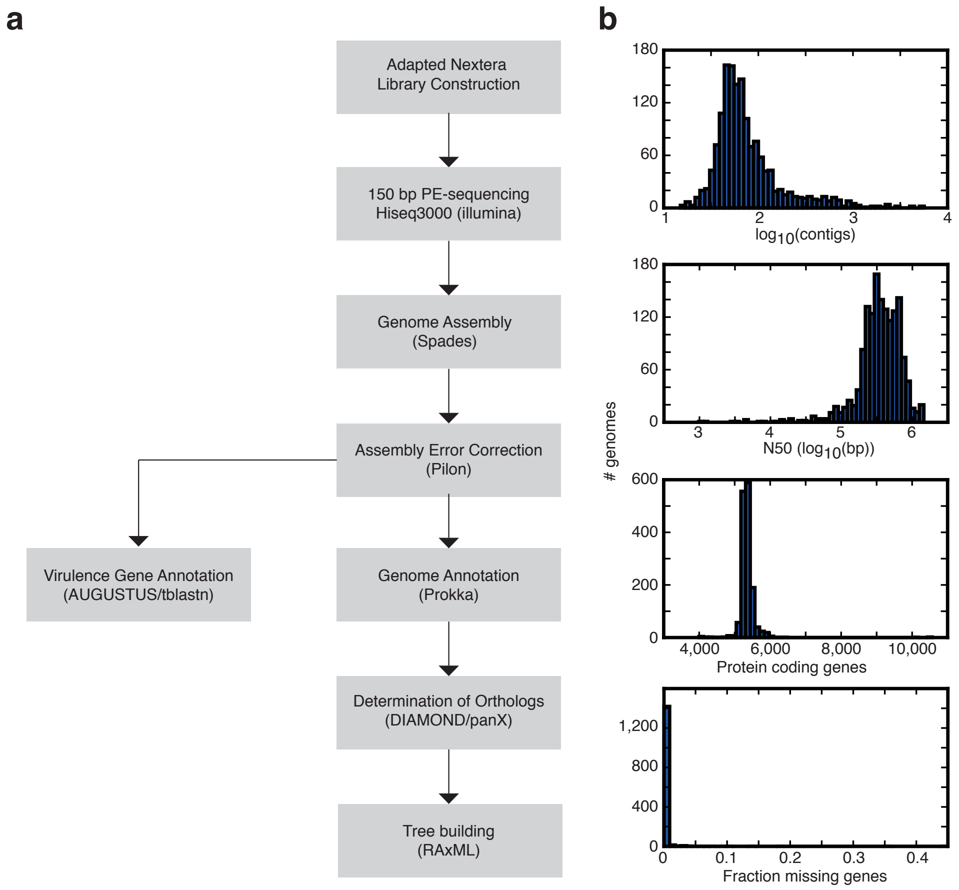

Fig. S3. De novo genome assembly of 1,524 strains sequenced in this study. Related to Fig. 4. Genomes were filtered for basic assembly quality (N50>25,000 bp, and >3,500 genes per genome). (a) The number of contigs and (b) N50, (c) the number of protein coding genes annotated, and (d) the percentage of genes predicted to be missing for each assembly are shown for the 1,524 genomes remaining after filtering.

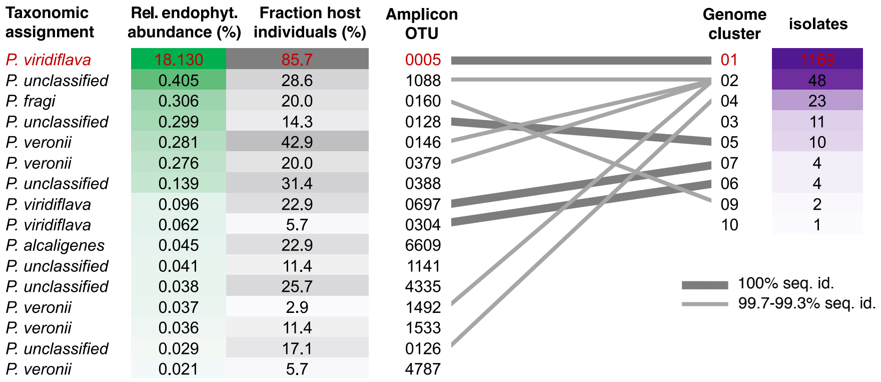

Fig. S4. Leaf-endophytic Pseudomonas diversity at Eyach site: overlap between amplicon sequencing and strain isolation data. Related to Fig. 5. Memberships in strain clusters identified by 99% sequence identity of the 16S rDNA v3-v4, either through amplicon sequencing (OTUs, left) or strain isolation (genomes, right). Matching groups (grey lines) were identified by comparing representative sequences for the amplicon OTUs/genome clusters. Even when considering data from different host individuals sampled at different times there is a good overlap between the two collections.

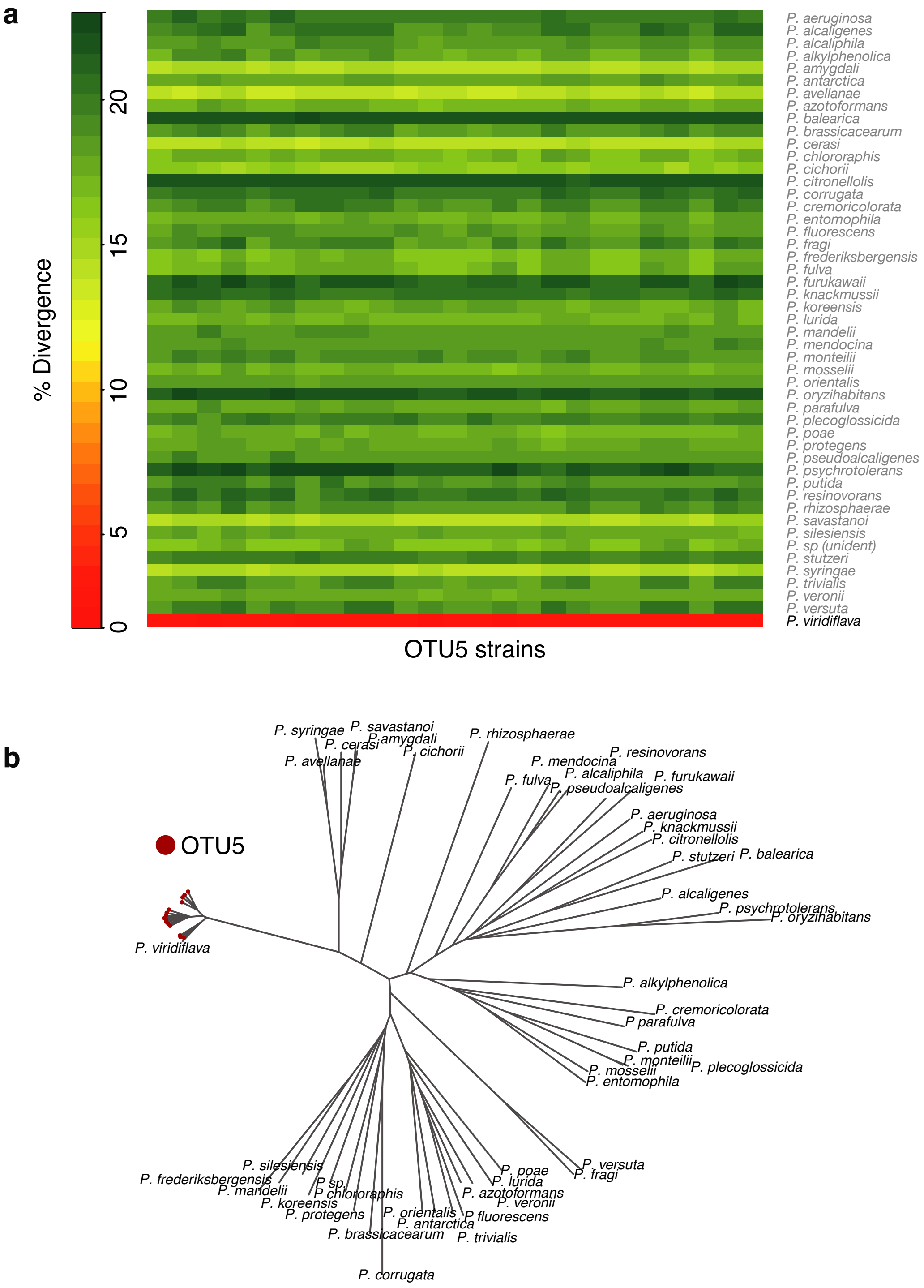

Fig. S5. Pairwise divergence between OTU5 and reference *Pseudomonas* genomes. Related to Fig. 1f. (a) Pairwise divergences between 25 randomly selected OTU5 genomes and a single representative reference genome from each of 51 *Pseudomonas* species were calculated using mash (Ondov et al., 2016). OTU5 strains share the greatest sequence similarity with *P. viridiflava*, differing on average by 2.6%. (b) A Neighbour-joining tree (Saitou and Nei, 1987) was constructed from the pairwise distances, again demonstrating the relatedness of OTU5 to *P. viridiflava*.

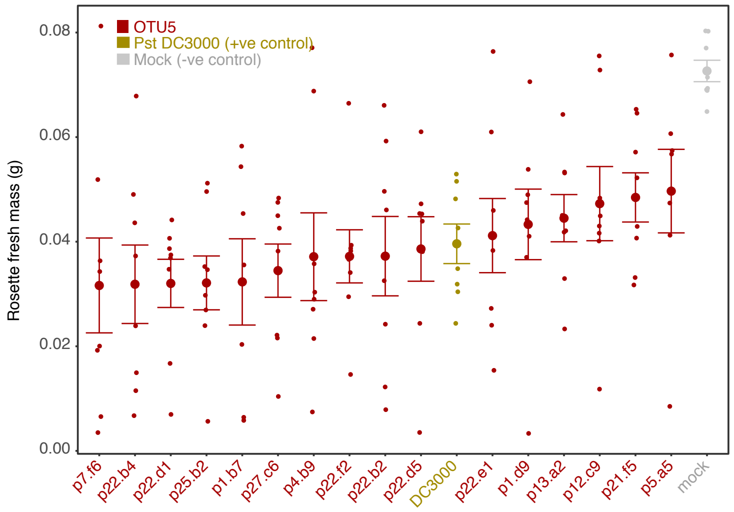

Fig. S6. Gnotobiotic trial with OTU5 strains. Related to Fig. 3c. 14-day old *A. thaliana* plants of accession Eyach 15-2 were drip-infected with different OTU5 strains. All tested strains significantly reduced plant growth (n=8 replicates per sample, Student's t-test, q-value <0.05).

Table S1. Collection locations and dates for all samples. Related to Fig. 1

Site	Latitude	Longitude	Sample type
EY	48.446111	8.781611	16S, metagenome, isolates
K6911	48.541278	9.0925	16S, metagenome
PFN	48.561087	9.109294	16S, metagenome
JUG	48.555722/48.556255	9.134833/9.135424	16S, metagenome, isolates
WH	48.506827	8.936418	16S
ERG	48.495362	8.809083	16S

Table S2. Genes annotated in plant-associated pathways and amino acid sequence of flg22 variants in OTU5. Related to Fig. 6. Top: The listed genes (right column) were custom-annotated in each of the genomes to ascertain the presence of the listed pathway (left column). Bottom: Left column lists the genome from which flg22 sequence was extracted. OTU5 encodes two major variants of flg22, one similar to a *P. floridae* genotype, and the other to a *P. viridiflava* genotype.

Pathway	Components
Coronatine	*cfa1, cfa2, cfa3, cfa4, cfa5, cfa6, cfa7, cfa8, cfa9, cmaA, cmaB, cmaC, cmaD, cmaE, cmaT*
Mangotoxin	*mboA, mboB, mboC, mboD, mboE, mboF*
Phaseolotoxin	*argK, amtA*
Syringomycin	*syrB1, syrB2, syrC, syrD, syrE, syrP*
Syringopeptin	*sypA, sypB, sypC*
Tabtoxin	*tblA, tabA, tabB*

Reference	flg22 Sequence
Pst DC3000	QRLSTGSRINSAKDDAAGLQIA
P. floridae	
(WP_083186033)	ERLSTGKKINTAADDAGGSITQ
OTU5 (649 isolates)	ERLSTGKKINTAADDAGGSITQ
P. viridiflava	
(WP_025995199)	SRLSSGLKVQNARDNVGVLSTI
OTU5 (670 isolates)	SRLSSGLKVQNARDNVGVLSTI
OTU5 (22 isolates)	SRLSSGLKVTNARDNVGVLSTI
Marinomonas sp.	QRLSSGKRINSAKDDAAGMQI
OTU5 (3 isolates)	QKLSSGKSITSSKDNAAGSQIA
